# Archaeal family B DNA polymerase facilitates lagging strand DNA replication in the *Thermococcales*

**DOI:** 10.1093/nar/gkag395

**Published:** 2026-05-04

**Authors:** Geraldy Lie Stefanus Liman, Marina D Black, Vladimir Potapov, Brett W Burkhart, Katherine Levine, Brenda Baker, Jaylin L Mandley, Sarah C Davidson, Andrew F Gardner, Thomas J Santangelo, Kelly M Zatopek

**Affiliations:** New England Biolabs, Inc., Ipswich, MA 01938, United States; Department of Biochemistry and Molecular Biology, Colorado State University, Fort Collins, CO 80523, United States; Department of Biochemistry and Molecular Biology, Colorado State University, Fort Collins, CO 80523, United States; New England Biolabs, Inc., Ipswich, MA 01938, United States; Department of Biochemistry and Molecular Biology, Colorado State University, Fort Collins, CO 80523, United States; New England Biolabs, Inc., Ipswich, MA 01938, United States; New England Biolabs, Inc., Ipswich, MA 01938, United States; Department of Biochemistry and Molecular Biology, Colorado State University, Fort Collins, CO 80523, United States; Department of Biochemistry and Molecular Biology, Colorado State University, Fort Collins, CO 80523, United States; New England Biolabs, Inc., Ipswich, MA 01938, United States; Department of Biochemistry and Molecular Biology, Colorado State University, Fort Collins, CO 80523, United States; New England Biolabs, Inc., Ipswich, MA 01938, United States

## Abstract

Replicative DNA synthesis is distributed among several DNA polymerases (DNAPs) in both Bacteria and Eukarya. The division of synthesis permits specialized activities of unique DNAPs to facilitate rapid replication in a continuous manner on the leading strand and discontinuous synthesis of the lagging strand with repetitive priming and maturation of Okazaki fragments. Many archaea encode two evolutionarily unrelated DNAPs, PolB and PolD, but how leading and lagging strand synthesis responsibilities are partitioned between each polymerase has yet to be resolved *in vivo*. In the model archaeon *Thermococcus kodakarensis*, Pol B is dispensable, demonstrating PolD can independently support replicative synthesis, but does not exclude the possibility that PolB normally participates in key aspects of DNA replication. Here, we combine phenotypic analyses of strains encoding variant DNAPs, high-throughput sequencing, and purified replication assays to establish that PolB actively facilitates lagging strand synthesis *in vivo*. Pol B outperforms PolD at strand displacement and Okazaki fragment maturation, demonstrating that specialized DNAPs share replicative synthesis duties in most Archaea. Our findings define a foundational division of labor between PolB and PolD in Archaea and establish the use of specialized replicative DNAP activities for strand-specific synthesis across all life.

## Graphical Abstract

**Graphical Abstract:**
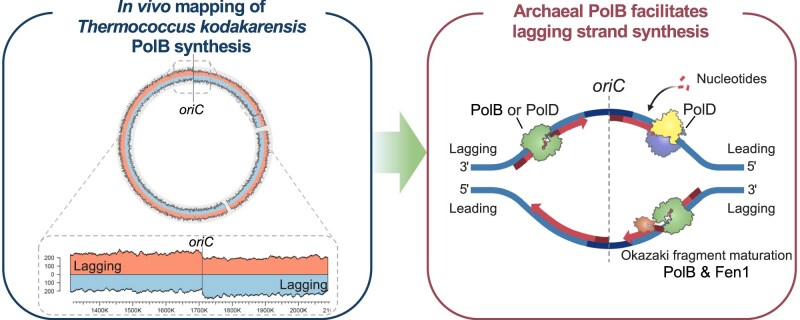

## Introduction

A division of labor amongst DNA polymerases (DNAPs) during discontinuous DNA replication is a hallmark of high-fidelity, rapid, and coordinated bidirectional DNA synthesis within Bacteria and Eukarya [1]. The use of specialized DNAPs with differential fidelities, processivities, 3′–5′ proofreading exonuclease activities, 5′–3′ nick-translation exonuclease activities, and strand-displacement synthesis activities tailors each DNAP to the unique requirements of leading and lagging strand synthesis. While faithful DNA replication is essential for accurate transmission of genetic information to daughter cells, at least two radically different frameworks for genomic replication have been established in extant Bacteria and Eukarya. One of the greatest differences lies in the identity, utilization, and coordination of replicative DNAPs responsible for chromosomal synthesis. In Bacteria, a family C DNAP, Pol III, carries out both leading and lagging strand synthesis, with Okazaki fragment maturation relegated to a family A DNAP, Pol I [2–4]. In Eukarya, in contrast, the vast bulk of replicative synthesis does not involve either family A or family C DNAPs and is instead driven by three family B DNAPs: Pol ⍺ extends RNA primers, while Pol ε and Pol δ carry out the bulk of leading and lagging strand synthesis, respectively [5, 6].

Indeed, DNAPs are currently divided into eight families based on amino acid sequence and three-dimensional structure: A, B, C, D, X, Y, reverse transcriptases (RTs), and archaeo-eukaryotic primases (AEP) [7, 8]. While families X, Y, and RTs minimally participate in genome replication, families A, B, C, and D encode DNAPs that synthesize the bulk of genomic DNA, and AEPs *de novo* synthesize DNA replication primers in Archaea and Eukarya [8, 9]. Further, while families A and C DNAPs participate in bacterial DNA replication and family B DNAPs participate in eukaryotic DNA replication, family D DNAPs are restricted to the archaea and are unlike other DNAP families, as their polymerase catalytic domain has evolved from the core fold of an RNA polymerase [10]. While PolD is encoded by most archaeal clades, it is notably absent from Crenarchaeota and has never been identified as the sole DNAP; instead, at least one (and often multiple) family B DNAPs are also encoded, raising longstanding questions regarding how archaeal polymerases partition replicative functions [11].

While many details of DNA replication in Bacteria and Eukarya are resolved, foundational parameters of DNA replication in Archaea remain comparatively underexplored. Fundamental aspects such as the full composition of the replisome, the requirements for and selective usage of origins, and even the roles of distinct DNAPs are open questions and are poorly defined for most archaeal clades. Little debate exists regarding the primary role of AEP in *de novo* synthesis of replication primers, although the mechanisms controlling AEP recruitment, processivity, fidelity, and nucleic acid context of the primer remain only partially characterized [12–14]. Conversely, the primary roles of PolB and PolD in archaeal replication have been debated since the identification of PolD, in part due to the diversity of archaeal replicative strategies [15–18]. For example, in *Thermococcus kodakarensis (T. kodakarensis), Thermococcus barophilus (T. barophilus), and Methanococcus maripaludis (M. maripaludis)*, PolB is non-essential, demonstrating that strains encoding only PolD are capable of supporting leading and lagging strand synthesis sufficient for viability and rapid growth [17, 19, 20]. In contrast, attempts to delete PolB1 in the halophilic archaeon *Haloferax volcanii* (*H. volcanii*) have been unsuccessful, implying essential roles for both PolB and PolD in genomic replication [21]. While many fundamental questions regarding archaeal DNA replication remain, none is perhaps more pressing than establishing which DNAPs are responsible for leading versus lagging strand synthesis.

In *Thermococcales*, Okazaki fragment maturation has been investigated through a variety of biochemical and genetic approaches, yet a unified mechanistic framework has not emerged. *In vitro* reconstitution studies using purified components from *T. kodakarensis* and related species have shown that both PolB and PolD can extend RNA–DNA primers and perform strand-displacement synthesis to generate 5′ flaps that are subsequently processed by Fen1 and sealed by archaeal DNA ligase [15, 16, 18, 22]. However, the extent and efficiency of strand displacement by PolB versus PolD have varied across studies, with reported activities differing depending on substrate design, protein composition, and reaction conditions. Collectively, these findings establish that the core enzymatic machinery required for Okazaki fragment maturation is present and functional, while leaving unresolved how strand displacement and fragment maturation are optimally executed *in vivo*.

*In vivo* genetic analyses in *T. kodakarensis* have shown that PolB, Fen1, GAN, and RNase HII are each individually dispensable under standard laboratory conditions, whereas PolD and PCNA are essential for viability, indicating that PolD alone can support lagging strand synthesis sufficient for growth [17, 23, 24]. However, dispensability does not preclude a specialized or facilitating role for PolB, particularly in strand-displacement and maturation reactions that are inherently discontinuous and kinetically complex. Notably, prior studies have neither quantitatively compared PolB- and PolD-mediated Okazaki fragment maturation nor directly linked polymerase-specific biochemical activities to strand-specific synthesis *in vivo*. Consequently, how lagging strand synthesis is optimized in *Thermococcales*, and whether distinct polymerases are preferentially deployed for fragment extension versus maturation, has remained unresolved.

Establishing which genomic sequences are primarily synthesized *in vivo* by a specific DNAP is non-trivial; the products of each DNAP are normally indistinguishable canonical DNA. Through selective genetic manipulations, individual DNAP activities *in vivo* can be altered such that the products of DNA synthesis by one DNAP are sufficiently differentiated to map and localize DNA synthesis to leading and/or lagging strands [25–28]. Such strategies have previously been employed to map Pol I to the lagging strand in *Escherichia coli* and the three eukaryotic PolBs to their respective roles in leading and lagging strand synthesis in yeast [28, 26]. Here, we employ a combination of genetic techniques to generate and phenotype strains of *T. kodakarensis* wherein an increased rate of ribonucleotide incorporation by variant PolB effectively marks the strand and regions of the genome synthesized *in vivo*. Utilizing these strains and subsequent high-throughput single-molecule sequencing, we demonstrate that PolB activities are largely restricted to the lagging strand, relegating leading strand synthesis to PolD. Consistent with a role in lagging strand synthesis, *in vitro* biochemical characterization of PolB and PolD reveals that PolB drives efficient strand displacement and Okazaki fragment maturation compared with PolD on a variety of putatively biologically relevant substrates. Together, these data suggest that Archaea, like Bacteria and Eukaryotes, encode specialized DNAPs that normally divide replicative synthesis tasks among unique enzymes. Thus, while Archaea encode information processing systems that, in general, more closely align with eukaryotic machinery, replication of their circular genomes appears to more closely mimic the division of replicative labor in Bacteria, with PolD, like Pol III, contributing the bulk of replicative synthesis while archaeal PolB fills an analogous role to bacterial Pol I in archaeal origin-dependent lagging strand DNA replication and Okazaki fragment maturation.

## Materials and methods

### Microbial growth and media conditions

All *T. kodakarensis* strains were cultivated following the previously described method [29]. Briefly, strains were inoculated anaerobically in artificial seawater media, which was supplemented with 5 g/l tryptone, 5 g/l yeast extract, 5 g/l sodium pyruvate, 2 g/l elemental sulfur (S°), a KOD1-vitamin mixture, and, optionally, 1 mM agmatine sulfate. Growth profiles of the parental strain (TS559) and the PolB-lacking strain (TS746) were generated from at least three independent biological replicates that were monitored at 85°C, 75°C, 65°C, and 55 °C by measuring optical density at 600 nm using a spectrophotometer. Data plotted in Microsoft Excel.

### *Thermococcus kodakarensis* strain constructions

Strains were constructed as previously described [29, 30]. Briefly, the deletions of the *polB* (TK0001) and *rnaseHII* (TK0805) loci were performed through homologous recombination, resulting in a *polB* deletion strain and a *polB/rnaseHII* double deletion strain, TS746 and TS747, respectively. The same genetic manipulation strategies were used to introduce the splicing-deficient mutant of TkRadA, which has a deletion of the Homing endonuclease (HEN) domain spanning residues 270 to 592 and includes 13 residues from *Pyrococcus horikoshii* RadA, specifically residues 273–285 *P.ho*. loop [31]. The genotypes of the deletion strains were confirmed via Oxford Nanopore MinION whole-genome sequencing at >100× coverage using TS559 as reference.

The TkoPolB complementation strains were constructed using the previously described pTS543 ectopic expression plasmid and previously determined exonuclease and steric gate mutations [30, 32, 33]. Briefly, pTS543 an encoding inteinless variant of *T. kodakarensis* PolBs also includes an expression cassette for TK0149, which encodes an arginine decarboxylase, restoring agmatine prototrophy as a selectable phenotype. Retention of the ectopic expression plasmids was confirmed via Plasmidsaurus whole-plasmid sequencing.

### Alkaline gel electrophoresis

Alkaline gel electrophoresis experiments used to qualitatively assess genome-wide ribonucleotide incorporation by PolB variants were conducted as previously described [34], with a few modifications. *T. kodakarensis* strains were grown to mid-exponential phase and harvested by centrifugation. Genomic DNA was isolated using the Monarch® Spin gDNA Extraction Kit (New England Biolabs, Inc.) with the Gram-positive Bacteria and Archaea protocol and quantified with the Qubit™ dsDNA BR Assay (Invitrogen™). A total of 2 µg of purified genomic DNA was treated with either 0.3 M NaOH or 0.3 M NaCl (negative control) at 55°C for 2 h to hydrolyze embedded ribonucleotides in the DNA. The samples were immediately mixed with 6× Purple loading dye (New England Biolabs, Inc.), loaded into a 1% alkaline-agarose gel (comprising 1% agarose, 1 mM EDTA, and 50 mM NaOH), and electrophoresed at 5 V/cm for 16 h at 4°C. The alkaline gel was then neutralized in 1 M Tris–HCl at pH 7.5 and 1.5 M NaCl; it was subsequently stained with SYBR Gold (Thermo Fisher Scientific) diluted to 1× in Tris–borate–EDTA buffer, which contains 89 mM Tris, 89 mM boric acid, and 2 mM EDTA. The stained gel was visualized using a Typhoon FLA 9500 (GE Healthcare). Image was analyzed and quantified with ImageQuant software and plotted with Microsoft Excel [34].

### Marker frequency analysis

The experimental procedure for marker frequency analysis was conducted as previously described and data from strains TS559 and AL015 are from our previous work, while those for strains AL029 and AM033 were generated for this study [31].

### RADAR-seq analysis of *T. kodakarensis* strains

Following growth, harvesting, and extraction of *T. kodakarensis* genomic DNA, as described above, RADAR-seq libraries were constructed as previously described for genome-wide ribonucleotide detection in *T. kodakarensis*, with a few modifications [28]. Genomic DNA was sheared into ∼5 kb fragments using Covaris g-tubes (Covaris, Woburn, MA) following the manufacturer’s protocol, followed by bead clean-up. PacBio libraries were constructed following the “Preparing multiplexed amplicon libraries using SMRTbell® prep kit 3.0” protocol using 1 µg input genomic DNA and the SMRTbell prep kit 3.0 and SMRTbell Adapter Index plate 96A to barcode each library. Following PacBio library construction, nick translation was performed as previously described using 9°N RNaseHII, Bst full-length DNA polymerase, Taq DNA ligase, NAD^+^, and a dNTP pool containing dTTP, dGTP, 6-methyl-dATP, and 4-methyl-dCTP, followed by a bead clean-up [28]. Libraries were quantitated using a Qubit Flex Fluorometer and sequenced on a Pacific Biosciences Sequel II Instrument, using the Sequel II Binding Kit 3.2, Sequel II Sequencing Kit 2.0, and Sequel II SMRTcell 8M tray using the ≥3 kb Amplicon Sequencing Application, 30 h movie time, and 140 pM on-plate loading concentration. Following sequencing, ribonucleotides were analyzed, quantitated, and mapped as previously described [28, 35]. Multiple biological replicates were performed for RADAR-seq analysis of each strain and were newly generated for this study (Table 1).

**Table 1.** Number of ribonucleotides detected in variant*T. kodakarensis* strains by RADAR-seq

Strain name	Genotype	Replicates	Total bases sequenced	rNMP per million bases sequenced	rNMP per genome
TS559	Wild type	3	1916070924	9 ± 3.4	38 ± 14
TS747	ΔPolB/ΔRNaseHII	3	1446412568	183 ± 26	761 ± 108
AL029	TS747 + pTS543-PolB^exo-/Y409V^	3	2659540985	218 ± 6	907 ± 25
AM030	TS747 + RadA^i^	2	1729671531	196 ± 8	815 ± 33
AM033	TS747 + RadA^i^ + pTS543-PolB^exo-/Y409V^	3	7812382999	261 ± 35	1085 ± 146

### Recombinant expression of *T. kodakarensis* DNA polymerases and Okazaki fragment maturation proteins

Genes encoding WT *T. kodakarensis* PolB (TK0001), proliferating cell nuclear antigen-1 (PCNA1; TK0535), flap endonuclease 1 (Fen1; TK1281), and DNA ligase (TK2140) were synthesized with N-terminal His-tags and individually cloned into a pET29a vector by Genscript (Piscataway, NJ). Genes encoding WT *T. kodakarensis* N-terminal His-tagged DP1 (TK1902) and DP2 (TK1903) PolD subunits, or N-terminal His-tagged PriS (TK1791) and PriL (TK1790) AEP primase subunits were synthesized and pairwise cloned into a pRSFDuet vector by GenScript. All clones were transformed into NiCo21 cells (New England Biolabs), plated on LB-KAN plates, and grown overnight at 37°C. A single colony of each clone was used to inoculate 1 L LB-KAN and grown overnight at 20°C, followed by transfer to 37°C and induction with 0.4 mM final IPTG at an OD_600_ of 0.4. Growth continued for 3 h at 37°C, and cells were harvested by centrifugation. For PolB, PolD, and PriSL, cells were resuspended in 200 ml of Buffer A (20 mM Tris–HCl, 0.3 M NaCl, pH 7.5) and lysed using a Shearjet HL60 cell homogenizer (Dyhydromatics), followed by heating to 80°C for 20 min. Heated lysate was pelleted by centrifugation, and supernatant was loaded onto a HiPrep DEAE column with Buffer A. Flow-through was loaded onto a 5 ml HisTrap column and eluted with a 0%–100% gradient to Buffer B (20 mM Tris–HCl, 0.3 M NaCl, 0.5 M imidazole, pH 7.5). Fractions were analyzed by SDS–PAGE to identify DNAP-containing fractions, which were pooled and loaded onto a HiLoad Superdex 200 16/600 column with Buffer A. Fractions were analyzed by SDS–PAGE, and DNAP-containing fractions were pooled and concentrated using 30 kDa Amicon cut-off filters, followed by dialysis into storage buffer (10 mM Tris–HCl, 100 mM KCl, 0.1 mM EDTA, 50% glycerol, pH 7.5). For PCNA, Fen1, and DNA ligase, cell pellets were resuspended in 50 ml of Buffer A and lysed using a multi-tipped Q500 sonicator (Qsonica). Lysate was heated to 80°C for 20 min, followed by centrifugation. Supernatant was incubated with 1 ml of NEBExpress Ni-NTA magnetic beads and incubated on a slow-tilt rotator at 4°C for 30 min. Lysate/beads were placed on a magnetic rack, supernatant was removed, and beads were washed twice with 25 ml of Buffer A, followed by elution with 2 ml of Buffer B. Eluted proteins were dialyzed with storage buffer and analyzed by SDS–PAGE for purity (Supplementary Fig. S1).

### *In vitro* strand displacement and Okazaki fragment maturation

To evaluate the *in vitro* strand displacement activities and quantify Okazaki fragment maturation of *T. kodakarensis* DNAPs, primer extension assays utilizing various DNA constructs and capillary electrophoresis (CE) were performed. A 50 nt 5ʹ-FAM labeled oligonucleotide primer (5ʹ/56-FAM/TAT AAC AGT TGA TTC CCA ATT CTG CGA ACG AGT AGA TTT AGT TTG ACC AT 3ʹ), a 150 nt complement (5ʹ AAT GCT ACT ACT ATT AGT AGA ATT GAT GCC ACC TTT TCA GCT CGC GCC CCA AAT GAA AAT ATA GCT AAA CAG GTT ATT GAC CAT TTG CGA AAT GTA TCT AAT GGT CAA ACT AAA TCT ACT CGT TCG CAG AAT TGG GAA TCA ACT GTT ATA 3ʹ), and a 90 nt 5ʹ-phosphate/3′-Cy3 oligonucleotide (5ʹ/5Phos/TCG CAA ATG GTC AAT AAC CTG TTT AGC TAT ATT TTC ATT TGG GGC GCG AGC TGA AAA GGT GGC ATC AAT TCT ACT AAT AGT AGT AGC ATT/3Cy3SP/3ʹ) were synthesized by IDT (Coralville, IO). A 90 nt 5ʹ-triphosphate/3ʹ-Cy3-labeled oligonucleotide (5ʹ P-P-P-TCG CAA ATG GTC AAT AAC CTG TTT AGC TAT ATT TTC ATT TGG GGC GCG AGC TGA AAA GGT GGC ATC AAT TCT ACT AAT AGT AGT AGC ATT-Cy3 3ʹ) and a 90 nt 5ʹ-monophosphate/3ʹ-Cy3-labeled RNA–DNA chimeric oligonucleotide (5ʹ P-rUrCrG rCrArA ATG GTC AAT AAC CTG TTT AGC TAT ATT TTC ATT TGG GGC GCG AGC TGA AAA GGT GGC ATC AAT TCT ACT AAT AGT AGT AGC ATT-Cy3 3ʹ) were constructed in-house as described below. Four DNA constructs were created, all at a final concentration of 1 µM in 1× DNA annealing buffer (10 mM Tris–HCl, 20 mM NaCl, pH 7.5). For construct 1, 50 nt 5ʹ-FAM primer and 150 nt complement were annealed in a 1:2 ratio. For constructs 2, 3, and 4, the 50 nt 5ʹ-FAM primer, 150 nt complement, and 90 nt downstream oligonucleotide were annealed in a 1:2:3 ratio, where construct 2 contains 90 nt 5ʹ-monophosphate all DNA downstream oligonucleotide, construct 3 contains 90 nt 5ʹ-triphosphate all DNA downstream oligonucleotide, and construct 4 contains 90 nt 5ʹ-monophosphate RNA–DNA chimeric downstream oligonucleotide. The ratio of 1:2:3 was chosen to ensure all 5ʹ-FAM primers contain a template, and all primer/templates contain a downstream oligonucleotide. Therefore, for three-piece Okazaki fragment maturation constructs, a fraction of the downstream Cy3 oligonucleotide remains unannealed and unreacted. For strand displacement assays, PolB and PolD at 12.5 nM final, or PriSL at 100 nM final, were incubated with 10 nM final construct 1, 2, 3, or 4 (for PolB and PolD) or just construct 2 (for PriSL), 100 µM final dNTPs in 1× Thermopol Buffer (20 mM Tris–HCl, 10 mM (NH_4_)_2_SO_4_, 10 mM KCl, 2 mM MgSO_4_, 0.1% Triton X-100, pH 8.8 at 25°C) in a 40 µl reaction at 50°C and 65°C for 1 min (PolB and PolD) or 3 min (PriSL), followed by quenching with equal volume 10 mM EDTA. Reactions were analyzed by CE as previously described [36]. For Okazaki fragment maturation assays, PolB and PolD (5 nM final) were incubated with 10 nM construct 2, 3, or 4, 100 µM dNTPs, 1 mM ATP, 200 nM PCNA, 100 nM DNA ligase, and 12.5 nM FEN1 in 1× Thermopol buffer in a 100 µl reaction for various time points (from 0.25–5 min) and quenched with an equal volume of 10 mM EDTA. For construct 3, Okazaki fragment maturation experiments were additionally performed at 2.5 nM and 10 nM final PolB and PolD to define optimal reaction conditions (Supplementary Fig. S2). Due to the formation of an abundance of strand displacement products for PolB at 10 nM final, 5 nM final was utilized for quantitave Okazaki fragment maturation assays. Reactions were analyzed by CE and quantified as previously described [36]. Importantly, quantification of reaction substrates and products was done utilizing the FAM channel and not the Cy3 channel, as all productive reactions are stoichiometrically constrained by the amount of FAM primer present.

### Construction of 5ʹ-triphosphate/3ʹ-Cy3 DNA and 5′-monophosphate/3′-Cy3 RNA–DNA 90 nt oligonucleotides

The 90 nt 5ʹ-triphosphate/3ʹ-Cy3 all DNA and 5′-monophosphate/3′-Cy3 RNA–DNA chimeric labeled oligonucleotides were constructed in-house. For 90 nt 5ʹ-triphosphate/3ʹ-Cy3, three DNA oligonucleotides were synthesized in-house: a 33 nt oligonucleotide 1 (5′-TCG CAA ATG GTC AAT AAC CTG TTT AGC TAT ATT-3′), a 57 nt 5′-monophosphate/3′-Cy3 oligonucleotide 2 (5′-P-TTC ATT TGG GGC GCG AGC TGA AAA GGT GGC ATC AAT TCT ACT AAT AGT AGT AGC ATT-Cy3-3′), and a 21 nt splint oligonucleotide 3 (5′-CCA AAT GAA AAT ATA GCT AAA-3′) each at a 1.0 µmol scale with DMT removed using an H-8 synthesizer from Sierra BioSystems, Inc., following standard phosphoramidite chemistry [37]. CPG supports 5′-dimethoxytrityl-2′-deoxythymidine (1000 A), 5′-dimethoxytrityl-N-benzoyl-2′-deoxyadenosine (1000 A), and 1-[3-(4-monomethoxytrityloxy)propyl]-3,3,3′,3′-tetramethylindocarbocyanine chloride-1′-propyl-3-O-succinoyl-long chain alkylamino-CPG, which were purchased from Glen Research. Ultramild base protected phosphoramidites, chemical phosphorylation reagent (CPR), and ancillary reagents for oligonucleotide synthesis were purchased from Glen Research. Ultramild DNA phosphoramidites and CPR were dissolved in dry acetonitrile to a concentration of 0.05 M. Following synthesis of oligonucleotide 1, 5′-triphosphorylation was achieved using 5-chloro-saligenyl-*N,N*-diisopropylphosphoramidite (ChemGenes) and bis(tetra-n-butylammonium) dihydrogen pyrophosphate (TCI Chemicals) as previously described [38], and deprotection was achieved via incubation with 1 mL ammonium hydroxide/methylamine at 65°C for 10 min. For oligonucleotides 2 and 3, deprotection was achieved via incubation with 1 mL concentrated ammonium hydroxide in water at 65°C for 2 h. The oligonucleotides were isolated and desalted by reverse-phase HPLC using an Agilent 1290 Infinity II and Waters XBridge Ost BEH C18, 300 Å, 2.5 µm column and characterized by LCMS using an Agilent AdvanceBio 6545XT LC/Q-TOF with an Agilent AdvanceBio Oligonucleotide 120 Å, 2.7 µm, 2.1 × 50 mm column. For the 5′-monophosphate/3′-Cy3 RNA–DNA chimeric labeled oligonucleotide, three oligonucleotides were synthesized by IDT: a 33 nt oligonucleotide 4 (5′/5Phos/rUrCrG rCrArA ATG GTC AAT AAC CTG TTT AGC TAT ATT 3′), a 57 nt 5′- monophosphate/3′-Cy3 oligonucleotide 5 (5′/5Phos/TTC ATT TGG GGC GCG AGC TGA AAA GGT GGC ATC AAT TCT ACT AAT AGT AGT AGC ATT/3Cy3SP/ 3′), and a 21 nt splint oligonucleotide 6 (5′ CCA AAU GAA AAU AUA GCU AAA 3′).

Isolated oligonucleotides were combined as follows: oligo 1 (1 equivalent), oligo 2 (0.96 equivalents), and oligo 3 (1.2 equivalents) or oligo 4 (1 equivalent), oligo 5 (0.96 equivalents), and oligo 6 (1.2 equivalents) were mixed and annealed in 367 µl of 10 mM Tris–HCl, 0.1 mM EDTA, and 50 mM NaCl (pH 8.0). Subsequently, 45 µl of 10× T4 DNA ligase buffer (NEB B0202), 8 µl of water, and 30 µl of T4 DNA ligase (NEB M0202) were added for a final volume of 450 µl and incubated at 16°C overnight. The reaction products were monitored by LCMS. For target 90 nt 5′-triphosphate/3′-Cy3, the oligonucleotide was isolated using a reverse-phase HPLC Xbridge Premier Oligo BEH C18, 130 Å, 2.5 μm, 4.6 × 50 mm column heated to 65°C to dissociate the splinted duplex. LCMS analysis of the oligonucleotide confirmed isolation of the desired full-length target 5-triphosphorylated oligonucleotide (theoretical mass: 28572.69 Da; observed deconvoluted mass: 28572.35 Da) (Supplementary Fig. S3). For target 5′-monophosphate/3′-Cy3 RNA–DNA chimeric oligonucleotide, following overnight ligation, 10 µl of USER enzyme (NEB M5505) was added and incubated for 1 h at RT, followed by clean-up with a NEB Monarch Spin PCR & DNA Clean-up Kit (T1130) for removal of the 21 nt split. The target oligo was isolated as described above by HPLC, and LCMS analysis was used to confirm isolation of the desired full-length target 5′-monophosphate RNA–DNA chimeric oligonucleotide (Theoretical Mass: 28631.7 Da; Observed Deconvoluted Mass: 28632.63 Da) (Supplementary Fig. S3).

## Results

### Introduction of PolB variants with increased ribonucleotide incorporation

In an effort to track *in vivo* DNA synthesis by PolB and answer a long-standing question regarding the division of labor amongst DNAPs in archaeal genome replication, we aimed to generate *T. kodakarensis* strains encoding mutational variant PolB enzymes that significantly increased ribonucleotide incorporation. Subsequent genome-wide ribonucleotide mapping via RADAR-seq would then define the genomic regions and strands synthesized by PolB [28]. Similar methodologies have been used to map *E. coli* DNA Pol I to the lagging strand and map the three eukaryotic replicative DNAPs (Pol ε, Pol ⍺, and Pol δ) to their respective roles on leading and lagging strands in yeast [26, 28]. To ensure retention of ribonucleotides incorporated by PolB into the genome of *T. kodakarensis*, it was critical to (i) increase the frequency of ribonucleotide incorporation of PolB by mutationally altering the rNTP versus dNTP discrimination residue termed the steric gate [39], (ii) eliminate the 3ʹ–5ʹ exonuclease activities of PolB in tandem such that ribonucleotide incorporations were not immediately removed, (iii) remove the two intein-encoding sequences from PolB to ensure that protein splicing did not compromise PolB activity, (iv) delete RNaseHII, the primary enzyme responsible for recognizing and initiating archaeal ribonucleotide excision repair [34], and (v) favor origin-dependent replication (ODR) such that defined regions of the genome can be assigned as leading and lagging strands [31]. Under optimal conditions, *T. kodakarensis* prefers to initiate DNA replication through a recombination-dependent replication (RDR) mechanism, resulting in a population of cells that effectively uses any and every position as an origin, obscuring the definition of leading and lagging strand assignment within a population [40, 31].

The non-essential nature of both PolB (TK0001) and RNaseHII (TK0805) in *T. kodakarensis* permitted the reintroduction of intein-less, mutational PolB variants of choice into a strain (TS747) lacking both PolB and RNaseHII (Supplementary Table S1 and Supplementary Fig. S4). The 3ʹ–5ʹ exonuclease activity of PolB is compromised by the introduction of a pair of substitutions (D141A and E143A) to the exonuclease active site. Given the nonessential nature of PolB, it was unsurprising that we were easily able to reintroduce an exonuclease-deficient, intein-less PolB variant into the native PolB locus on the genome of strain TS747, generating strain AL006 (Supplementary Table S1). We were surprised, however, that exhaustive efforts to introduce any exonuclease-deficient, intein-less PolB variant that also compromised the steric gate through mutation of the steric gate residue Y409 into the native PolB locus of strain TS747 were not tolerated, given that PolB is dispensable in *T. kodakarensis*. The intolerance of these mutations indicates that PolB performs an essential function when present, pointing to a more substantial role than initially assumed (Supplementary Table S1). The relatively close proximity of Y409 to one of the extein-intein boundaries drove initial concerns that substitution of Y409 was compromising intein splicing, but difficulties in strain construction persisted even when the intein-encoding regions of PolB were removed. Even when we seemingly succeeded in reintroduction of desirable PolB variants into the native PolB locus of strain TS747, we noted that these rarely recovered single isolates always carried spontaneous second-site suppressor mutations within the PolB encoding sequence that abolished PolB activity. Thus, while PolB is non-essential, repeated confirmation that reintroduction of select PolB variants into the genome is inviable immediately implied that PolB had a function in replicative synthesis that could be replaced by PolD but not replaced by a PolB that lacked exonuclease activity and that was compromised for ribonucleotide discrimination. Consistent with eukaryotic systems, where catalytically impaired or steric-gate mutant alleles of Polδ and Polε confer replication defects and lethality not observed in simple deletions, our data imply that defective polymerases may be more disruptive than their absence [41–45].

To circumvent our inability to reintroduce PolB variants that permitted increased ribonucleotide incorporation into the genome of strain TS747, we instead reintroduced PolB via ectopic expression plasmids. In contrast to the severe limitations encountered with genomic reintroductions of PolB variants, ectopic expression allowed complementation in most instances [30]. While some steric gate compromized PolB variants were not tolerated (see Supplementary Table S1) even when ectopically expressed, we were successful in generating TS747-derived strains ectopically expressing intein-less PolB lacking exonuclease activity (AL027; PolB^exo−^), and two strains encoding PolB lacking exonuclease activity with either a Y409L or Y409V substitution in the PolB steric gate (AL028; PolB^exo-/Y409L^ and AL029; PolB^exo-/Y409V^).

**Figure 1.**
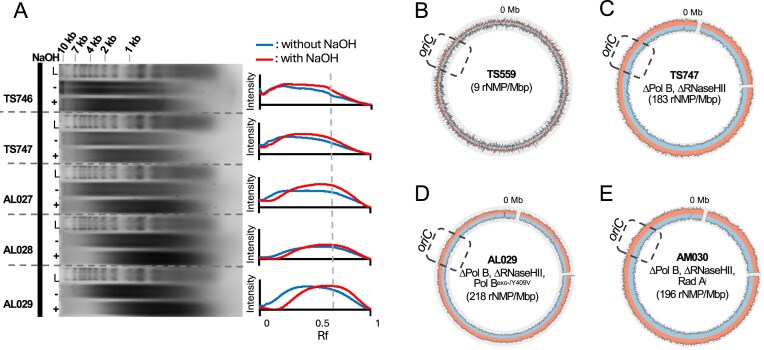
(**A**) Alkaline gel electrophoresis of genomic DNA from *T. kodakarensis* strains to qualitatively assess ribonucleotide incorporation level, with the highest abundance of ribonucleotides observed in AL029 (ΔPolB/ΔRNaseHII + pTS543 PolB^exo-/Y409V^). Gray vertical line placed at the center of the AL029 NaOH profile. L corresponds to ladder. RADAR-seq plots showing even distribution of ribonucleotides mapped to *T. kodakarensis* strains (**B**) WT (TS559), (**C**) ΔPolB/ΔRNaseHII (TS747), (**D**) ΔPolB/ΔRNaseHII + pTS543 PolB^exo-/Y409V^ (AL029), and (**E**) ΔPolB/ΔRNaseHII/RadA^i^ (AM030). Gray box outlines the predicted origin of replication.

To assess whether reintroduced PolB activities result in increased ribonucleotide incorporations—and thus demonstrate that PolB minimally participates in DNA synthesis *in vivo*—we purified genomic DNAs from a strain lacking PolB (TS746), a strain lacking PolB and RNaseHII (TS747), and strains lacking PolB and RNaseHII that were ectopically expressing PolB variants (AL027, AL028, and AL029) for separations through denaturing agarose gels following alkaline hydrolysis (Fig. 1A). Alkaline treatment of genomic DNAs results in spontaneous cleavage at sites of ribonucleotide incorporation, reducing fragment size and increasing electrophoretic mobility. In full support of increased ribonucleotide accumulation upon deletion of RNaseHII and reintroduction of PolB variant enzymes, the DNA migration patterns following NaOH versus NaCl exposure yielded faster migrating DNA fragments for strains TS747, AL027, AL028, and AL029, whereas no significant differences in fragment size were observed for strain TS746. Strain AL029, which encodes PolB^exo-/Y409V^, appears to have the greatest accumulation of genomic ribonucleotides based on alkaline hydrolysis of bulk genomic DNA.

While denaturing alkaline gels provide a qualitative metric of total rNMP incorporation frequencies between strains, RADAR-seq provides a quantitative, strand- and position-specific technique to map ribonucleoside monophosphate (rNMP) incorporations on a genome-wide level. RADAR-seq has been previously used to map the location of *in vivo* DNAP synthesis in *E. coli* [28]. Within the RADAR-seq workflow, ribonucleotides embedded within genomic DNA are converted into patches of methylated deoxynucleoside monophosphates (dNMPs) that are then identified using PacBio SMRT sequencing. Counting and mapping methylated patches defines the abundance, strand, and genomic positions of ribonucleotides incorporated into the genome [28].

Matching current and previous bulk denaturing-alkaline electrophoresis metrics of rNMP incorporations, RADAR-seq analyses of our parental strain (TS559) revealed only low levels of rNMP incorporations, while deletion of PolB in combination with RNaseHII (TS747) led to a >20-fold increase in ribonucleotides that were uniformly distributed on both strands and across the genome (Fig. 1B and C and Table 1) [28]. Previous RADAR-seq analysis of RNaseHII deletion alone in *T. kodakarensis* showed a >50-fold genome-wide increase in ribonucleotides. The comparatively smaller (∼20-fold) increase observed with the combined PolB and RNaseHII deletion suggests that PolB is responsible for a substantial fraction of aberrant ribonucleotide incorporation and likely contributes to DNA replication [28]. The blank regions observed in the RADAR-seq plots correspond to the proviral regions TKV1 (TK0073–TK0105) and TKV2 (TK0381–TK0421). These elements are present in the WT TS559 genome but are absent in the deletion strains, consistent with the known mobility of these proviral elements [46, 47]. When RADAR-seq was applied to map rNMP incorporations in AL029 (PolB^exo-/Y409V^), we observed a very modest further increase in total rNMPs incorporated per genome, suggesting that the ectopically expressed PolB variant is an active participant in replication and/or repair across the entire genome (Fig. 1D and Table 1). We observed no enrichment of rNMP incorporations to a specific strand or genomic location in AL029 (Fig. 1D and Supplementary Fig. S5). This lack of enrichment to any strand or genomic region was anticipated given AL029 is grown under optimal laboratory conditions and is thus employing RDR, not ODR. While ODR initiates from a single origin and thus defines leading and lagging strands in a bidirectional orientation stemming from the origin, RDR initiates at random genomic positions. When mapping genomic replication on the population level, RDR does not establish congruent leading or lagging strands to any specific area of the genome [21, 31, 40, 48].

### PolB activity maps to the lagging strand in origin-dependent strains

We can tip *T. kodakarensis* genomic DNA replication in favor of ODR over RDR in strains wherein the mature protein levels of the key recombination protein RadA are reduced by limiting the efficiency of spontaneous intein splicing from the precursor RadA protein, as visualized using marker frequency analysis (Supplementary Fig. S6) [49, 31]. Our parental strain TS559 displays a flat MFA profile indicative of RDR, whereas strain AL015—which reduces mature RadA levels *in vivo* just ∼2-fold—displays a sigmoidal MFA profile with the apex defining the bioinformatically defined *ori* sequence in the *T. kodakarensis* genome (Supplementary Fig. S6A and B). Matching expectations, strain AL029 (ΔPolB, ΔRNaseHII + PolB^exo-/Y409V^) also displays a flat MFA profile, confirming that attempts to map PolB activities within this strain to the leading or lagging strand (when considering the population rather than individual cells) are impossible due to the dominance of RDR (Supplementary Fig. S6C).

We therefore generated strains wherein we combined the RadA-modified intein-encoding sequence from strain AL015 with the variant ectopically expressed PolB^exo-/Y409V^ in an RNaseHII and PolB deletion background. We first incorporated the modified RadA intein allele (RadA^i^) into strain TS747 (ΔPolB/ΔRNaseHII), thereby generating strain AM030 (ΔPolB/ΔRNaseHII/RadA^i^) (Supplementary Table S1 and Supplementary Fig. S4). Introduction of a modified RadA allele into our strains did not change the levels or regions of rNMP incorporation (Fig. 1E and Table 1). AM030 served as the host for our ectopic plasmid encoding PolB^exo-/Y409V^, generating strain AM033 (Supplementary Table S1 and Supplementary Fig. S4). Strain AM033 was predicted to shift the replicative strategy from RDR to ODR and permit mapping of any strand or genomic location-specific activities of variant PolB enzymes *in vivo*. MFA of DNA recovered from strain AM033 in exponential phase clearly confirmed a shift in replicative strategy to ODR from RDR, with a sigmoidal profile defining the *ori* (Supplementary Fig. S6D).

**Figure 2.**
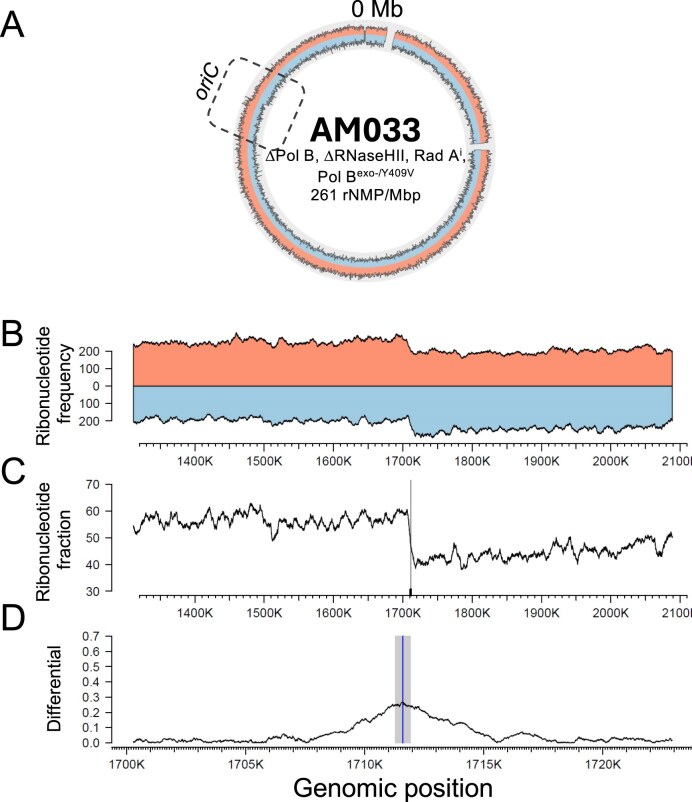
RADAR-seq plot for (**A**) AM033 (ΔPolB/ΔRNaseHII/RadA^i^/pTS543 PolB^exo-/Y409V^). Gray box outlines the predicted origin of replication. (**B**) Ribonucleotide frequency, obtained from RADAR-seq analysis of AM033, near the putative origin of replication, where a characteristic switch in ribonucleotides is observed. (**C**) The fraction of ribonucleotides found on the top strand is displayed in the genomic region surrounding the putative origin of replication (vertical line). (**D**) Precise genomic location in which ribonucleotide frequency switches, as determined by locating the position with the maximal value of differential [28]. Gray area corresponds to the predicted origin of replication.

Given the origin utilization observed in strain AM033, we next performed RADAR-seq on strain AM033 to map the abundance, strand, and positions of genomic ribonucleotide incorporations (Fig. 2A and Supplementary Fig. S7). Consistent with strains TS747 and AL029 that also lack RNaseHII, we again observed high ribonucleotide incorporation levels in strain AM033 (261 rNMP/Mbp). Most excitingly, while we did not observe any obvious strand bias to the accumulation of ribonucleotides for any other strain, we observed an obvious and well-defined strand bias of ribonucleotides stemming bidirectionally from the predicted origin (genomic location of 1711287–1711921) in AM033 (Fig. 2A and B). RADAR-seq established increased levels of rNMP incorporations on the lagging strand that switches between the positive and negative strands exactly at the presumptive *ori* (Fig. 2B), as would be predicted for bidirectional DNA replication from a single origin. This pattern of replication polarity at origins has been previously observed in Bacteria and Eukarya and is now observed also in Archaea [26, 28]. Further analysis of the crossover point of ribonucleotide incorporation from the positive to negative strand correlates with the genomic position of the presumptive origin at base pair resolution (Fig. 2B–D). This precise mapping further defines the origin and for the first time establishes an *in vivo* role for PolB in lagging strand synthesis during genomic replication in *T. kodakarensis*.

### Archaeal DNAPs retain dramatically different strand displacement synthesis activities

**Figure 3.**
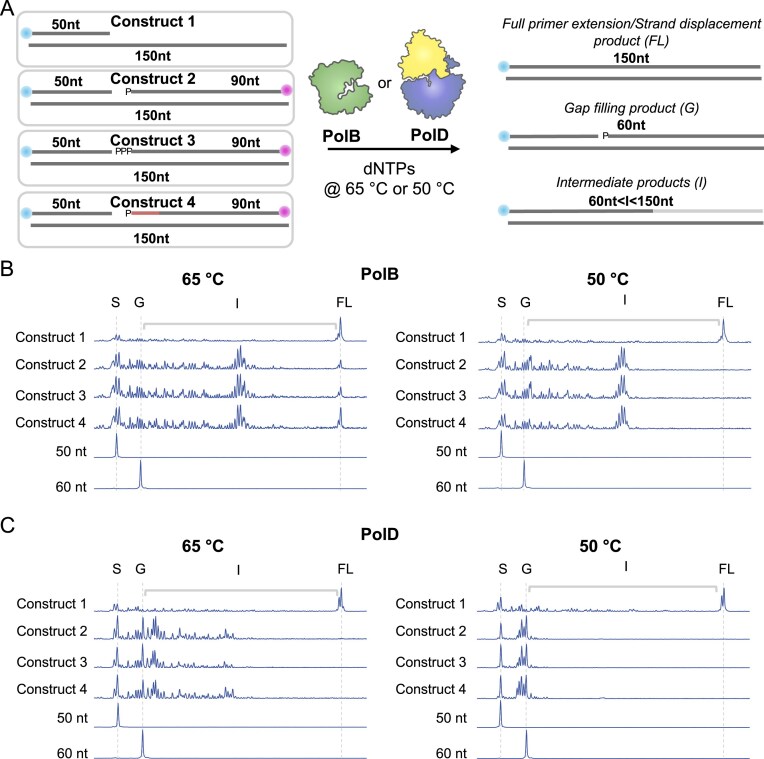
*In vitro* CE strand displacement assay. (**A**) Schematic of the CE strand displacement assay, where a 5ʹ-FAM-labeled primer is annealed to a 150 nt template (construct 1) or with a 90 nt downstream oligo that contains a 5ʹ-monophosphate all DNA (construct 2), 5ʹ-triphosphate all DNA (construct 3), or 5ʹ-monophosphate RNA–DNA chimera (construct 4) and is incubated with PolB or PolD and dNTPs at 65°C or 50°C for 1 min and quenched with EDTA followed by CE analysis. CE traces displaying the DNAP (construct 1) and strand displacement activities (construct 2, 3, or 4) of PolB (**B**) and PolD (**C**) at 65°C or 50°C, along with a 50 nt substrate control and a 60 nt gap-filling product control. CE traces display a 50 nt substrate (S), a 60 nt gap-filling product (G), strand displacement intermediates (I), and full-length extension products (FL).

Nick translation activities, driven by 5ʹ–3ʹ exonuclease activities of bacterial Pol I, support the removal of primase-incorporated ribonucleotides during Okazaki fragment maturation in Bacteria [50]. In contrast, the known and presumptive replicative DNAPs in Eukarya (Pol δ and Pol ε) and Archaea (PolB and PolD), respectively, lack 5ʹ–3ʹ exonuclease activities and instead rely on strand displacement activity to initiate Okazaki fragment maturation during lagging strand synthesis. Establishing a role for PolB in lagging strand synthesis in *T. kodakarensis* suggests that PolB may retain specialized activities that better facilitate Okazaki fragment maturation than PolD. We thus aimed to directly compare the strand displacement activities of Tko PolB, PolD, and the AEP PriSL under identical experimental conditions using a series of increasingly idealized biologically relevant substates (Fig. 3 and Supplementary Figure S8).

Following expression and purification of each *T. kodakarensis* DNAP (Supplementary Fig. S1A), we performed primer extension activity assays on four DNA constructs: a 50 nt 5ʹ-FAM labeled primer annealed to a 150 nt template (construct 1); an identical scaffold that included a downstream all DNA 90 nt deoxyoligonucleotide containing either a 5ʹ-monophosphate (construct 2) or 5ʹ-triphosphate (construct 3); or an RNA–DNA chimera 90 nt oligonucleotide containing a 5ʹ-monophosphate (construct 4), which leaves a 10 nt gap between upstream and downstream strands (Fig. 3A). Current evidence in *Thermococcales* supports the presence of 5′ ribonucleotides in replication primers *in vivo*, despite *in vitro* biochemical data indicating that PriSL preferentially synthesizes largely deoxyribonucleotide primers; moreover, the distribution of 5′ mono- and triphosphorylated termini remains unclear [12–14]. Following incubation with *T. kodakarensis* PolB, PolD, and PriSL at both 65°C and 50°C, extension products were resolved using CE (Fig. 3B and C and Supplementary Fig. S8). Due to poor strand displacement activity of *T. kodakarensis* PriSL even at high concentrations (10-fold over substrate) and with extended incubation time (3 min), we ruled out PriSL as an active participant in *T. kodakarensis* Okazaki fragment maturation (Supplementary Fig. S8).

Both PolB and PolD efficiently extended the 50 nt primer to the end of the 150 nt template of construct 1 in 1 min at both 65°C and 50°C, as evident by the appearance of the full-length product (Fig. 3B and C). However, under all conditions tested, PolB exhibited more efficient strand displacement activity compared to PolD. For example, at 65°C, we observe appreciable amounts of full-length product produced by PolB in all constructs, with no full-length product and minimal strand-displacement intermediates observed for PolD under the same experimental conditions (Fig. 3B and C). In fact, reduced temperature (50°C) completely abolished PolD strand displacement activity on all constructs, suggesting that PolD is unable to actively facilitate strand displacement and that the apparent strand displacement activity of PolD at higher temperatures may simply reflect PolD extensions reliant on thermal strand denaturation. Taken together, consistent with a biological role in lagging strand synthesis and Okazaki fragment maturation *in vivo*, PolB has more efficient strand-displacement activities compared to PolD *in vitro*.

### Archaeal PolB but not PolD facilitates robust Okazaki fragment maturation *in vitro*

**Figure 4.**
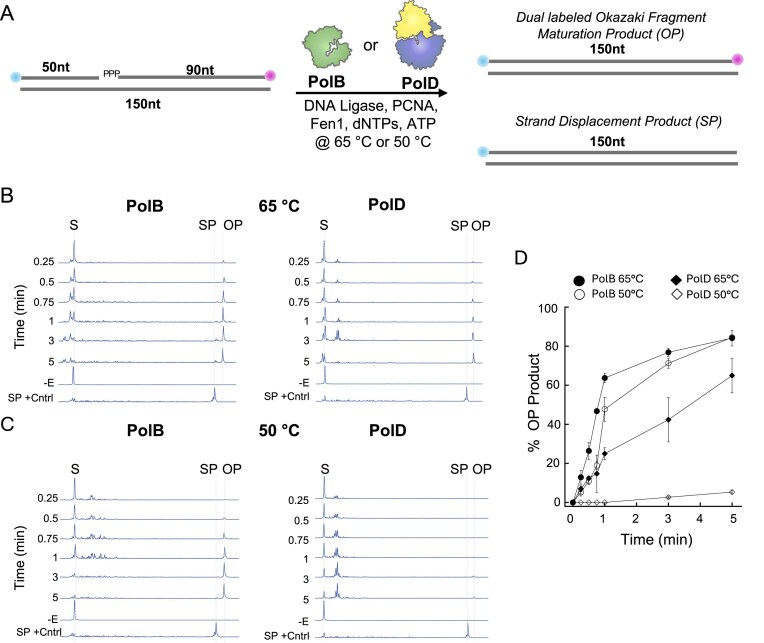
*In vitro* CE Okazaki fragment maturation assay. (**A**) Schematic of the CE-based Okazaki fragment maturation assay where a 50 nt 5ʹ-FAM primer and a 90 nt 5ʹ-triphosphate 3ʹ-Cy3 downstream oligo are annealed to a 150 nt template and incubated with FEN1, PCNA, DNA ligase, dNTPs, ATP, and either PolB, PolD, or both at 65°C or 50°C for various time points and quenched with EDTA followed by CE analysis. CE traces from Okazaki fragment maturation time courses for PolB and PolD at (**B**) 65°C or (**C**) 50°C, where OP = Okazaki fragment maturation product, SP = strand displacement product, and SP+control is a CE trace for polymerase strand displacement reaction. (**D**) % Product of OP formed during Okazaki fragment maturation time courses. Error bars represent mean ± SD of experimental triplicates.

The capacity for strand-displacement DNA synthesis is necessary but insufficient to mature Okazaki fragments. Maturation is a highly coordinated event carried out by a complex of (minimally) PCNA, a flap endonuclease activity (Fen1 in archaea and eukarya), and DNA ligase, which work in concert with a DNAP to process and join adjacent Okazaki fragments. To assess the ability of PolB and PolD to facilitate Okazaki fragment maturation, we additionally purified recombinant *T. kodakarensis* Fen1, PCNA, and DNA ligase (Supplementary Fig. S1B). While PCNA has no direct enzymatic activity, its addition is known to stimulate the activities of each archaeal DNAP, while Fen1 is required for 5ʹ end processing, and DNA ligase joins the two Okazaki fragments in an ATP-dependent manner [16, 23, 24]. Here, we incubated PolB or PolD with PCNA, Fen1, and DNA ligase at 65°C and 50°C with our various constructs (constructs 2, 3, and 4) to observe how differing 5′ ends and temperature affect Okazaki fragment maturation *in vitro* (Fig. 4 and Supplementary Figs S9 and S10). Construct 2 is all DNA and contains a 5′ monophosphate at the gap and therefore does not require DNAP strand displacement activity for maturation. Construct 3 is all DNA and contains a 5ʹ-triphosphate at the gap, and construct 4 contains a 5′-monophosphate followed by an RNA–DNA chimera, and both require DNAP strand displacement synthesis and Fen1 processing for DNA ligation to occur. Importantly, all constructs contain a 3ʹ-Cy3 label on the downstream strand that enables us to track the predicted co-migration of the FAM and Cy3 fluorophores that will only occur in correctly matured Okazaki fragment products (Supplementary Fig. S11). Alternatively, when strand displacement synthesis displaces the downstream 3ʹ-Cy3-labeled oligonucleotide, we observe a FAM-extended primer that is easily distinguished from our co-migrating dual-fluorophore Okazaki fragment maturation product in our CE traces (SP +Cntrl in Fig. 4 and Supplementary Figs S9 and S10). Due to the large excess of the Cy3-labeled strand that was required to ensure all constructs contain a downstream strand (see the “Materials and methods” section), quantitation was performed utilizing the FAM channel. Here, for all constructs tested, we observed efficient Okazaki fragment maturation product formation in the presence of PolB over PolD at both 65°C and 50°C (Fig. 4 and Supplementary Figs S9 and S10). For both PolB and PolD, the most efficient maturation was observed for the 5ʹ-monophosphate all DNA construct 2, followed by 5ʹ-triphosphate all DNA construct 3, and finally 5ʹ-monophosphate RNA–DNA chimera construct 4, which correlates with the amount of strand displacement required for DNA ligation to occur. While construct 2 requires no strand displacement synthesis, construct 3 requires minimal strand displacement synthesis due to the presence of just the 5ʹ-triphosphate, and construct 4 requires the displacement of, at minimum, the six 5ʹ terminal ribonucleotides prior to ligation. Importantly, at lower temperatures we observe minimal to undetectable Okazaki fragment maturation in the presence of PolD, which correlates with the observed poor strand displacement ability of PolD at lower temperatures (Fig. 4C and D and Supplementary Figs S9 and  S10C and D). Collectively, our *in vitro* biochemical data demonstrate that PolD exhibits limited strand displacement activity, suggesting that while PolD can participate in Okazaki fragment maturation, it does so inefficiently, while PolB displays robust strand displacement activity and facilitates robust Okazaki fragment maturation.

### PolB strains are cold-sensitive

**Figure 5.**
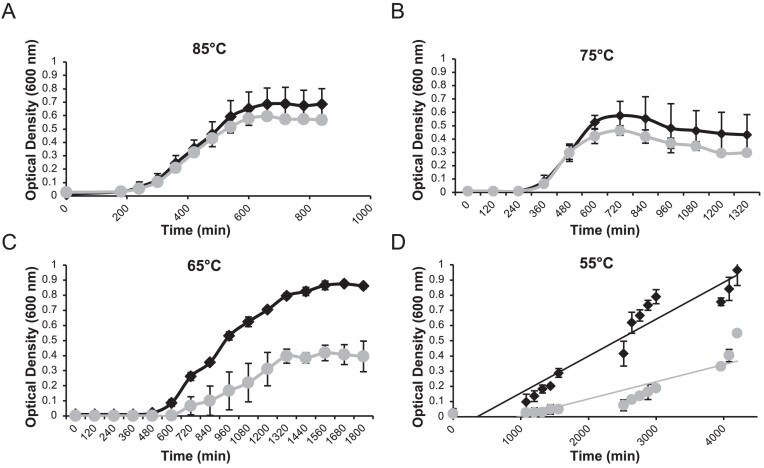
Growth curves of WT (TS559) (♦) and ΔPolB (TS746) (●) *T. kodakarensis* strains at (**A**) 85°C, (**B**) 75°C, (**C**) 65°C, and (**D**) 55°C.

The poor strand displacement synthesis activities of PolD *in vitro* are seemingly in conflict with the requirement for strand displacement synthesis during lagging strand replication *in vivo* and the lack of significant growth phenotype when PolB is deleted from *T. kodakarensis*. The temperature dependence of PolD-mediated strand displacement synthesis *in vitro*, and the potential that PolD extensions are primarily driven by thermal denaturation rather than true PolD-mediated strand displacement activities, suggest that while strains encoding only PolD might thrive at high growth temperatures, these same strains may show more impactful phenotypes when grown at reduced temperatures that would reduce thermal denaturation of paired DNAs. While PolB deletion results in DNA-damage sensitive strains, a ΔPolB (TS746) strain grows nearly identical to the parental strain (TS559) at its optimal growth temperature of 85°C (Fig. 5) but displays a significant growth phenotype at lower growth temperatures, with the most significant phenotype observed at 55°C (Fig. 5A–D and Supplementary Fig. S12) [17]. In support of PolD effectively substituting for strand-displacement synthesis activities normally carried out by PolB on the lagging strand at 85°C, the clear fitness negative growth impacts of strain TS746 at lower temperatures are suggestive that temperature reductions impact PolD capacities for key lagging strand synthesis activities. Taken together with our *in vivo* genetics/sequencing and *in vitro* biochemical experiments, these key activities are likely strand displacement synthesis for efficient Okazaki fragment maturation that takes place on the lagging strand.

## Discussion

**Figure 6.**
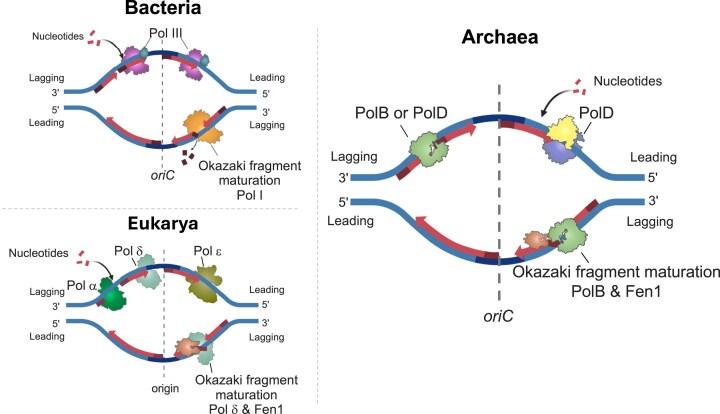
Current models of chromosomal DNA replication across domains of life. In Bacteria, DNA replication is carried out by DNA polymerase III, which synthesizes both the leading and lagging strands, while DNA polymerase I removes RNA primers and completes Okazaki fragment maturation. In Eukarya, Pol α-primase extends RNA primers, Pol ε primarily synthesizes the leading strand, and Pol δ synthesizes the lagging strand and participates in Okazaki fragment maturation. In Archaea, replication is centered on PolD, which performs leading-strand synthesis, while lagging-strand synthesis is proposed to be carried out by PolD, PolB, or both, and Okazaki fragment maturation is mediated by PolB.

Multiple DNAPs are encoded in archaeal genomes, most typically PolD and PolB, but the relative contributions of each archaeal DNAP to replicative synthesis *in vivo* have not been rigorously established. The complexities of bidirectional replicative synthesis are partitioned to unique DNAPs in both Bacteria and Eukarya, hinting that specialized roles for PolD and PolB might be expected in Archaea. While PolD alone can support viability in the model hyperthermophilic marine archaeon *T. kodakarensis*, a customary function of PolB in replication may be the norm, with the activities established for PolB *in vitro* suggesting a potential role in lagging strand synthesis. Through extensive genetic efforts, here we mapped the activities of variant PolB enzymes in *T. kodakarensis* to the lagging strand (Fig. 2). This foundational result, that PolB normally participates in lagging strand synthesis and Okazaki fragment maturation, argues that the evolutionary retention of PolB in all archaeal genomes divides replicative synthesis among specialized DNAPs, thus matching the partitioning of replicative activities in Bacteria and Eukarya (Fig. 6). Interestingly, eukaryotic replicative PolBs appear to be hybrids of archaeal PolD and PolB [51]. They retain the PolB-type polymerase and exonuclease catalytic cores but have also acquired the PolD DP1 subunit, referred to as subunit 2, which plays a critical regulatory and structural role in Pol α, Pol δ, and Pol ε [52]. Thus, whereas Archaea rely on PolD for the bulk of genome replication and PolB for Okazaki fragment maturation, the DP2 catalytic subunit of PolD was likely replaced by the PolB-type enzyme during evolution, leading eukaryotes to employ PolBs for both leading- and lagging-strand DNA synthesis [53].

Our mapping procedures were reliant on modified PolB enzymes that incorporate greater numbers of rNMPs into the genome and RADAR-seq-based mapping technologies to quantify and define the locations and strands of ribonucleotide incorporation. We note that while PolB is nonessential, reintroduction of steric gate variant PolB enzymes that were known to radically impact ribonucleotide discrimination was not tolerated at the native PolB locus nor through ectopic expression. Due to the high temperature at which *T. kodakarensis* thrives and the lability of embedded ribonucleotides within genomic DNA, it is likely that the accumulation of such leads to DNA breaks and high genome instability. Further, the intolerance of select PolB variants is reminiscent of phenotypes observed in yeast for the lagging strand-specific eukaryotic DNAP, Pol δ, where mutations to the POL3 catalytic subunit are often detrimental, causing lethality, replication failure, chromosome instability, or severe damage [54–56]. The inability to generate many strains encoding mutationally variant PolB enzymes suggests that PolB normally participates in DNA replication and can be substituted for PolD when absent, but when PolB fidelity and activities are sufficiently impacted, cells cannot survive.

The PolB variant that was viable via ectopic vector expression, PolB^exo-/Y409V^, increased ribonucleotide incorporation levels that permitted mapping of PolB activities to the lagging strand, but this result does not define PolB as the *sole* lagging strand DNAP. Archaeal Okazaki fragments are quite small (just ∼150 bp), and our rNMP mapping does not allow us to distinguish between whether PolB is responsible for the bulk of lagging strand synthesis, akin to eukaryotic Pol δ, or whether PolB normally only assists with Okazaki fragment maturation, akin to bacterial Pol I [5, 14]. Indeed, a balance between viability and increased ribonucleotide incorporation is at play and is not archaeal-specific. When similar ribonucleotide mapping techniques were employed to track yeast Pol δ and Pol ε synthesis, strains in which the steric gate residues of these DNAPs were mutated had severe growth defects, and therefore adjacent residues were mutated to promote cellular fitness but still permit sufficient ribonucleotide incorporation for mapping [26, 45]. While steric gate-specific mutations of the DNAPs that physically replicate the bulk of the yeast genome were highly fitness negative, a yeast strain encoding a Pol α steric gate variant (Y869A) was viable without significant fitness impacts, suggesting the limited synthesis activities of Pol α during yeast DNA replication were readily tolerated. In *E. coli*, mutation of the steric gate residue of Pol I, E710, led to a poorly growing cell line, and thus the adjacent residue, I709, was mutated to glycine to permit increased ribonucleotide incorporation while maintaining cell viability [28]. Thus, we interpret our results for archaeal PolB more in line with a significant role in lagging strand synthesis, wherein loss of ribonucleotide discrimination would be more impactful, rather than a role for PolB in simply facilitating lagging strand maturation, wherein loss of ribonucleotide discrimination would be perhaps more easily tolerated. Higher resolution studies will be necessary to fully discriminate between a role for PolB in the bulk of lagging strand synthesis versus a more limited role for PolB in lagging strand maturation.

While we demonstrate a role for PolB in lagging strand synthesis during origin-dependent replication, we were unable to specifically track PolB activity during RDR (i.e. strand AL029). This limitation likely arises from the stochastic nature of initiation during RDR, where RadA-mediated strand invasion occurs at random, undefined genomic regions. Although RADAR-seq detects ribonucleotides at the single-molecule level, a population-level analysis across many genomes is required to generate a signal sufficient to reveal strand-specific ribonucleotide incorporation. While we currently lack a clear understanding of archaeal RDR initiation and progress to fully matured genomes, studies of recombination-mediated replication in eukaryotic systems utilize the RadA homolog RAD51 to initiate strand invasion at random double-strand breaks, with DNA synthesis proceeding through both leading and lagging strand synthesis, suggesting that archaeal PolB may similarly contribute to lagging strand synthesis during RDR [57]. Indeed, in strain AL029, encoding PolB^exo-/Y409V^, ribonucleotide accumulation is evenly distributed across the entire genome, suggesting that PolB is actively participating in DNA replication and repair during RDR.

Further, while the results here showed clear origin utilization at the presumptive oriC and localized PolB to lagging strand synthesis by observance of ribonucleotide accumulation on opposite positive and negative strands at the oriC, we did not detect a secondary strand switch in ribonucleotide accumulation opposite *oriC* that would indicate site-specific termination of DNA replication. This likely reflects several factors, including the absence of a bacterial-like Tus/Ter termination system in archaea and the continued activity of RDR alongside origin-dependent replication in strain AM033 [58]. It is therefore plausible that archaeal DNA replication terminates in a manner similar to eukaryotes, not at defined termination sites, but stochastically through replisome collisions [59]. Consequently, our findings extend an understanding not only of archaeal replication initiation and the division of labor among replicative DNAPs but also of how archaeal replication termination occurs.

Archaeal family B DNAPs, such as Vent, and Deep Vent, are regularly used for PCR amplifications and thus require robust strand displacement activities as would be necessary for lagging strand synthesis and Okazaki fragment maturation [60]. The relative efficiency of PolB and PolD strand displacement synthesis is debated, with *in vitro* biochemical studies reporting conflicting and opposite results [15, 16, 18]. To resolve the efficiencies of both DNAPs under identical conditions and with a variety of substrates that have been implicated as relevant lagging-strand substrates, we purified and measured strand-displacement synthesis activities of *T. kodakarensis* PolB and PolD (Fig. 3). While PolD struggles, or completely fails to display strand displacement synthesis activities, we readily observe both robust strand displacement and Okazaki fragment maturation activity by PolB (Figs 3 and 4 and Supplementary Figs S9 and S10). Further, the efficiency of PolB-mediated strand displacement activity confounded our ability to analyze Okazaki fragment maturation experiments, as PolB strand displacement products competed with Okazaki fragment maturation products, even in the presence of excess DNA ligase to drive strand ligation (Supplementary Fig. S2). Our temperature-dependent probing of PolD suggests a lack of innate strand displacement capabilities and instead implies that high temperature facilitates DNA breathing, and strand fraying enables PolD to extend the primer through the downstream deoxyoligonucleotide. While PolD depends on the replicative MCM helicase to unwind the leading-strand DNA *in vivo*, Okazaki fragment maturation occurs separately from the replisome and independently of the replicative helicase. The temperature dependence of PolD strand-displacement synthesis activities *in vitro* is also displayed *in vivo* by demonstrating a cold-sensitive phenotype in strains lacking PolB (Fig. 5). Our results suggest that PolD is inefficient at joining Okazaki fragments at reduced temperatures, thus leading to a slower rate of genome duplication and slower cell division. Given that stain TS746 utilizes RDR, not ODR, the temperature-sensitive phenotype further supports a normal role for PolB in RDR-mediated genomic replication.

In addition to specified biochemical activities that support tailored DNAP roles, specific roles for DNAPs are often supported by structural features that confer distinct activities. As an example, the bacterial Pol III holoenzyme synthesizes the bulk of leading and lagging strand DNA and is tightly associated with the replicative DnaB helicase to ensure encapsulation within the replisome [61]. In contrast, bacterial Pol I is not tightly associated with the replisome and largely functions independently of such to join lagging strands after replisome passage. Similarly, both eukaryotic Pol α and ε are held tightly within the replisome via a helicase binding domain present on subunit 2, while Pol δ, which performs lagging strand synthesis, lacks a tight association to the replicative helicase [62–64]. Specialized structural features and associations of archaeal DNAPs also support specialized roles for PolB and PolD *in vivo*. PolD, but not PolB, is recovered in replisome purifications from *T. kodakarensis* [17]. The DP1 exonuclease subunit of the heterodimeric archaeal PolD is a structural homolog to subunit 2 of eukaryotic replicative DNAPs that facilitates a tight association of PolD to the replisome via a homologous helicase binding domain [65]. PolB lacks any specific domains or features to associate with the replicative helicase, and further, PolB houses identical 3ʹ–5ʹ exonuclease and polymerase domains, DnaQ and Klenow-like, respectively, to bacterial Pol I [66].

While the core challenge of faithfully duplicating genetic material is universal, Bacteria, Eukarya, and Archaea have evolved distinct frameworks to accomplish genome replication. Bacteria rely on a streamlined system centered around family C Pol III and efficient Okazaki fragment maturation by family A DNA Pol I, enabling rapid and continuous replication (Fig. 6). Eukaryotes, with their larger and more complex genomes, employ a highly coordinated network of specialized family B DNAPs, chromatin remodelers, and RNA primer processing enzymes to manage replication within the context of chromatin and cell cycle regulation. Archaea, though often considered a blend of bacterial simplicity and eukaryotic complexity, exhibit unique adaptations, including a replication system centered around family D PolD and, as displayed in this work, efficient Okazaki fragment maturation by family B PolB to meet the demands of their diverse genome organization and replication strategies, including polyploidy and diverse modes of replication initiation. These evolutionary variations underscore that while the goal of DNA replication is conserved and all rely upon specialized DNAPs, the molecular strategies to achieve it are remarkably diverse, offering critical insight into both the evolution of cellular life and the mechanisms that preserve genomic integrity.

## Supplementary Material

gkag395_Supplemental_File

## Data Availability

RADAR-seq PacBio sequencing data and MFA Illumina sequencing data pertaining to this study has been deposited in the Sequencing Read Archive under accession number PRJNA1354854.
