# Supplementary material for: Archaeal family B DNA polymerase facilitates lagging strand DNA replication in the Thermococcales
Source: Nucleic Acids Res. 2026 May 4;54(8):gkag395. doi: 10.1093/nar/gkag395 (PMC13136892; doi:10.1093/nar/gkag395)
Supplement: gkag395_Supplemental_File [file gkag395_supplemental_file.pdf]

Supplemental Table 1. All attempted and achieved strains for this study

| Strain Name | Genotype | Attempted via Recombination driven genetics (RDG) | Successful RDG[a] | Attempted via Ectopic expression vector (EEC) | Successful EEC[a] |
|---|---|---|---|---|---|
| TS559 | ΔTK0149*, ΔTrpE::PyrF, ΔTK0664**, ΔPyrF | - | - | - | - |
| TS746 | TS559 + ΔPolB | ✓ | ✓ | - | - |
| TS747 | TS746 + ΔRNaseHII | ✓ | ✓ | - | - |
| AL008 | TS746 + PolB-Exo- | ✓ | ✓ | - | - |
| AL006 | TS747 + PolB-Exo- | ✓ | ✓ | - | - |
| AL027 | TS747 + pTS543-PolB-Exo- | - | - | ✓ | ✓ |
| AL028 | TS747 + pTS543-PolB-Exo-Y409L | - | - | ✓ | ✓ |
| AL029 | TS747 + pTS543-PolB-Exo-Y409V | - | - | ✓ | ✓ |
| AM030 | TS747 + RadA[i] | ✓ | ✓ | - | - |
| AM033 | AM030 + pTS543-PolB-Exo-Y409V | - | - | ✓ | ✓ |
| - | TS747 + PolB-Exo-Y409A | ✓ | NV | | |
| - | TS747 + PolB-Exo-Y409F | ✓ | NV | | |
| - | TS747 + PolB-Exo-Y409S | ✓ | NV | | |
| - | TS747 + PolB-Exo-Y409G | ✓ | NV | | |
| - | TS747 + PolB-Exo-Y409L | ✓ | NV | | |
| - | TS747 + PolB-Exo-Y409V | ✓ | NV | | |
| - | TS747 + PolB-Exo-A485L | ✓ | NV | | |
| - | TS746 + PolB-Exo-Y409A | ✓ | NV | | |
| - | TS746 + PolB-Exo-Y409F | ✓ | NV | | |
| - | TS746 + PolB-Exo-Y409S | ✓ | NV | | |
| - | TS746 + PolB-Exo-Y409G | ✓ | NV | | |
| - | TS746 + PolB-Exo-Y409L | ✓ | NV | | |
| - | TS746 + PolB-Exo-Y409V | ✓ | NV | | |
| - | TS746 + PolB-Exo-A485L | ✓ | NV | | |

Supplemental Table 1 Continued. All attempted and achieved strains for this study

| Strain Name | Genotype | Attempted via Recombination driven genetics (RDG) | Successful RDG | Attempted via Ectopic expression vector (EEC) | Successful EEC |
|---|---|---|---|---|---|
| - | TS747 + pTS543-PolB-Exo-Y409A | | | ✓ | NV |
| - | TS747 + pTS543-PolB-Exo-Y409F | | | ✓ | NV |
| - | TS747 + pTS543-PolB-Exo-Y409S | | | ✓ | NV |
| - | TS747 + pTS543-PolB-Exo-Y409G | | | ✓ | NV |
| - | AM030 + pTS543-PolB-Exo-Y409A | | | ✓ | NV |
| - | AM030 + pTS543-PolB-Exo-Y409F | | | ✓ | NV |
| - | AM030 + pTS543-PolB-Exo-Y409S | | | ✓ | NV |
| - | AM030 + pTS543-PolB-Exo-Y409G | | | ✓ | NV |

[a] NV reflects strains that were not viable

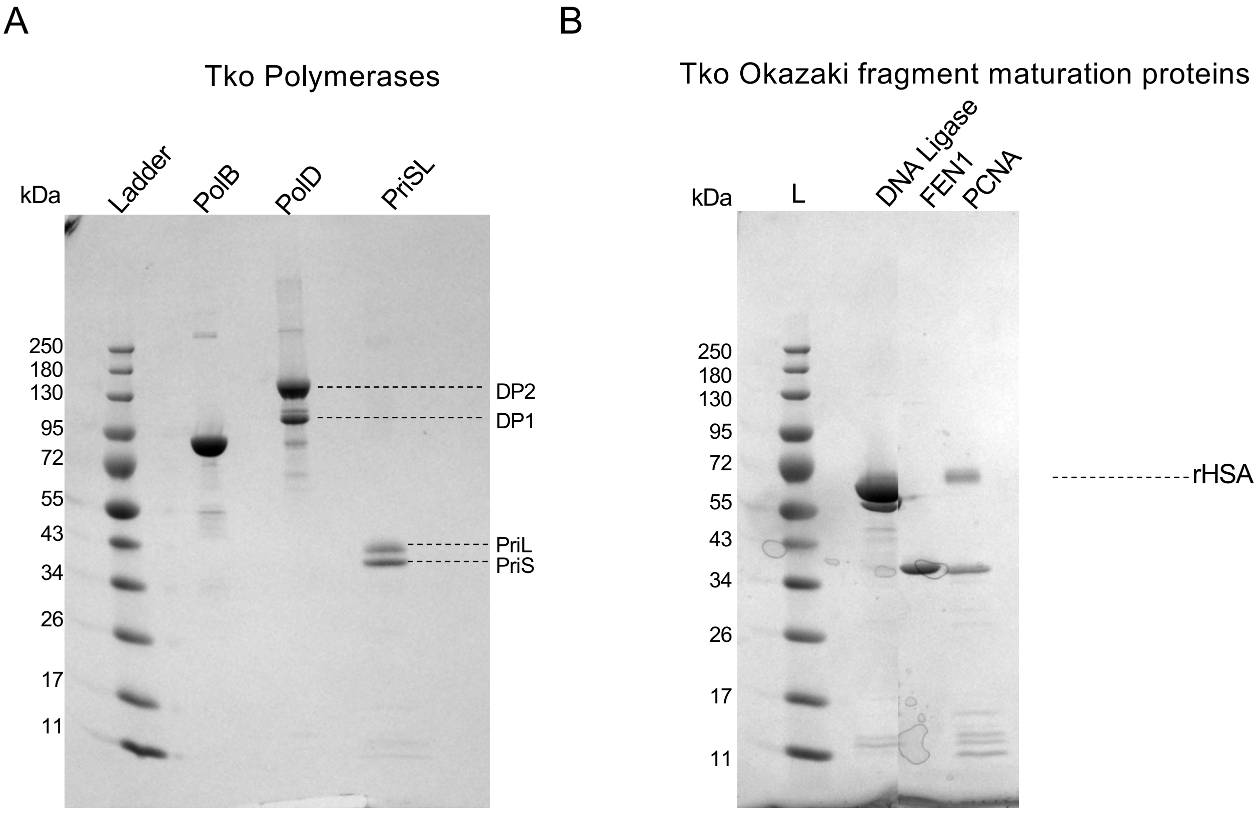


Supplemental Figure 1. SDS-PAGE gels of purified *T. kodakarensis* (A)DNA polymerases and (B)Okazaki fragment maturation proteins. PCNA was dialyzed into storage buffer + recombinant human serum albumin (rHSA).

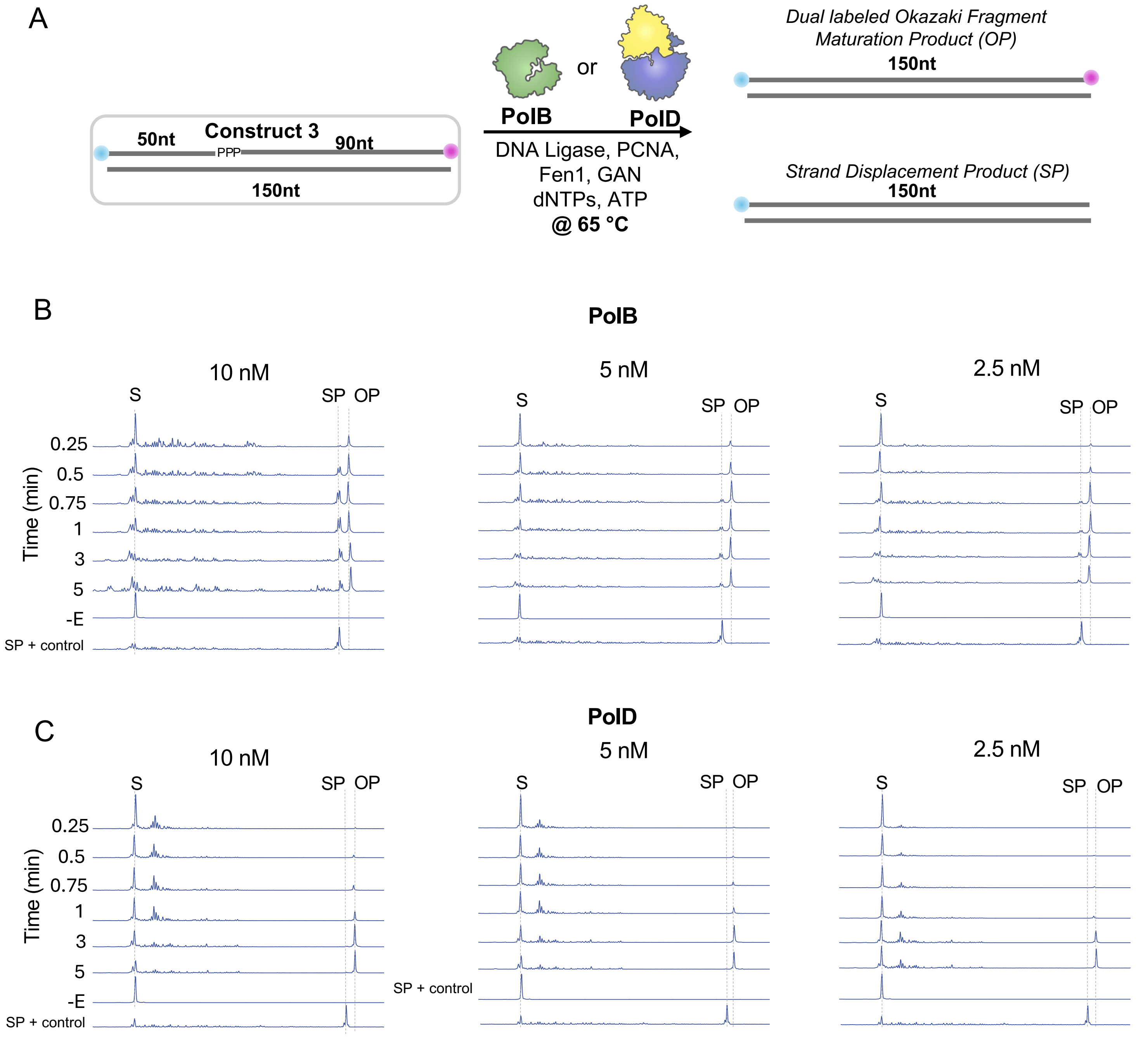


Supplemental Figure 2. DNA polymerase concentration dependence on Okazaki fragment maturation. (A) Schematic of the starting substrate and products formed during Okazaki fragment maturation. (B) PolB concentration dependence on formation of Okazaki fragment product (OP) versus strand displacement product (SP). (C) PolD concentration dependence on formation of Okazaki fragment maturation product (OP).

A

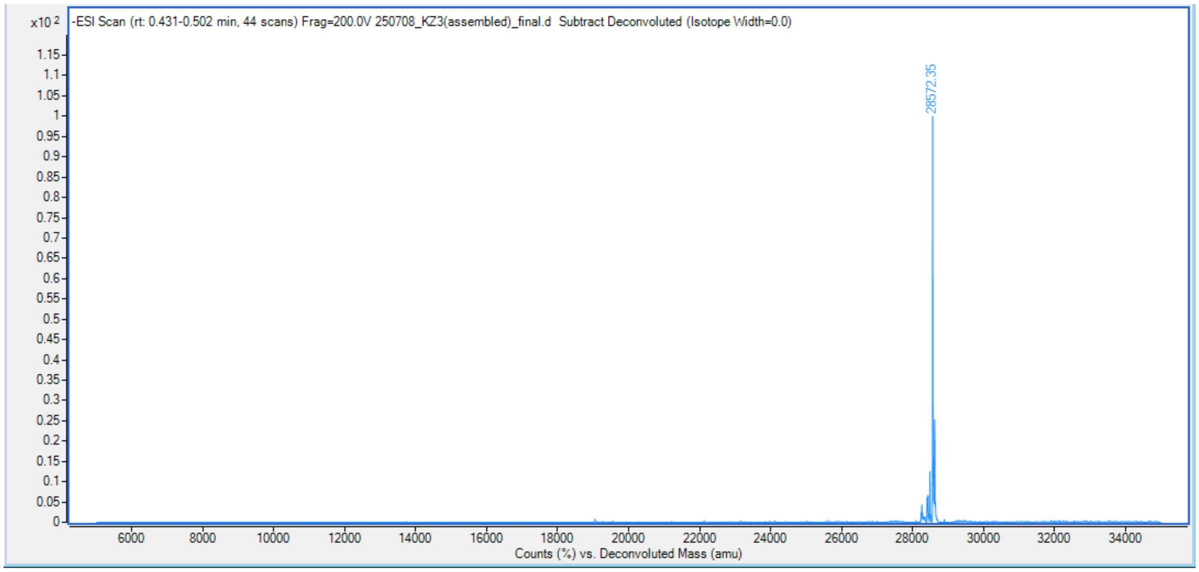


B

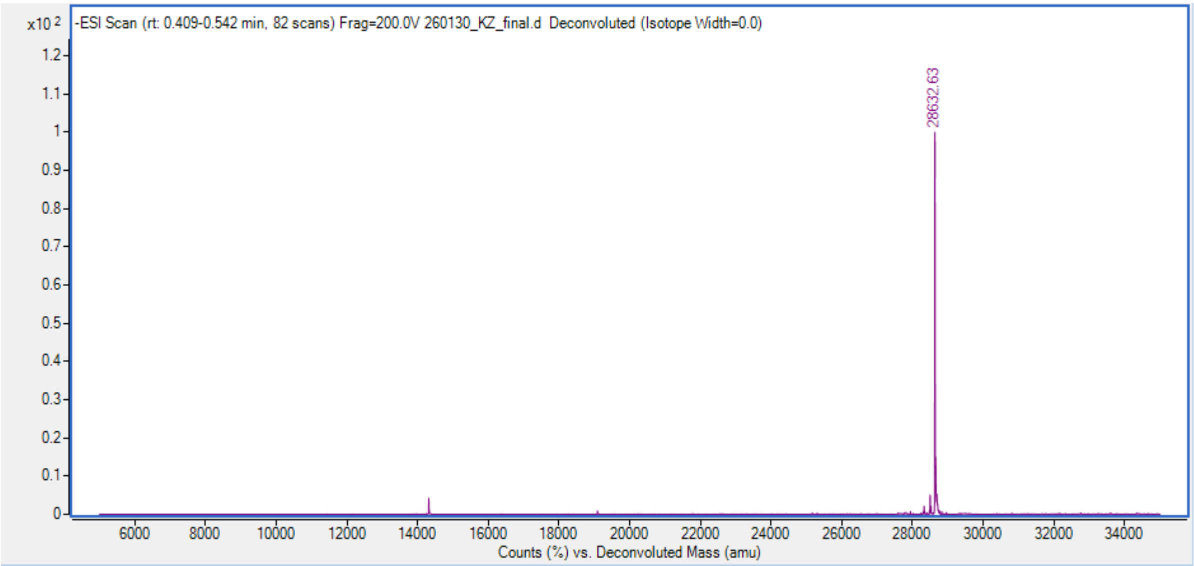


Supplemental Figure 3. Mass Spectrometry trace of full length (A) 90 nt 5'-triphosphate/3'-Cy3 all DNA oligonucleotide synthesized in-house with a theoretical mass of 28572.69 Da and an observed deconvoluted mass of 28572.35 Da and (B) 90 nt 5'-monophosphate/3'-Cy3 RNA-DNA chimera constructed in-house with a theoretical mass of 28631.7 Da and an observed deconvoluted mass of 28632.63 Da.

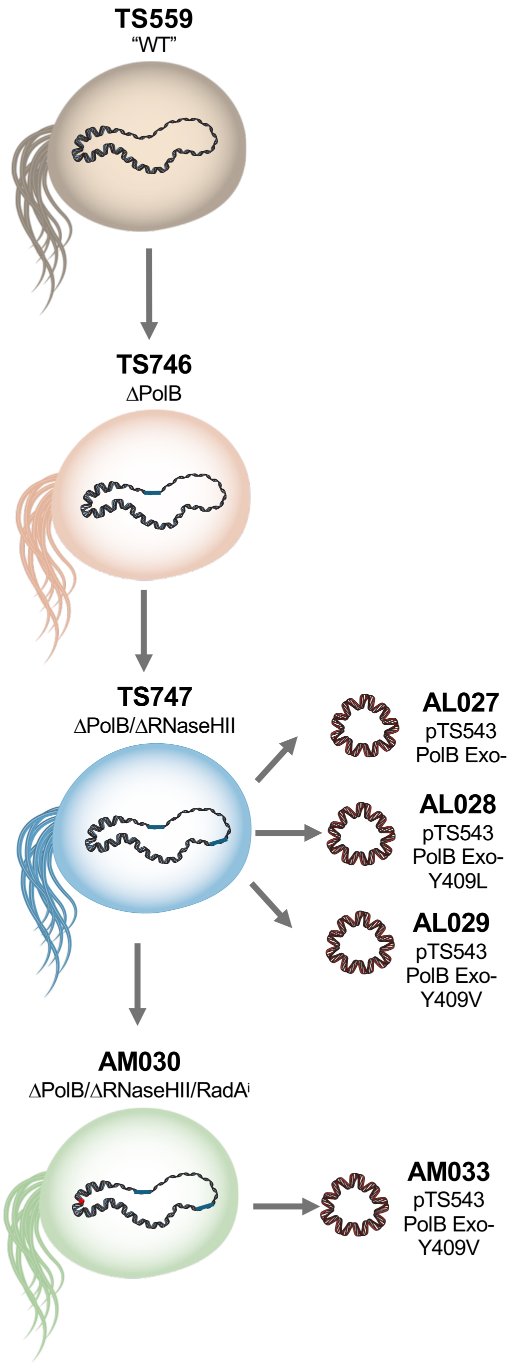


Supplemental Figure 4. (A) Schematic of *T. kodakarensis* strain construction. TS559 "WT" was first deleted of PolB (TS746), followed by deletion of RNaseHII (TS747). Variant PolBs were reintroduced via an ectopic expression vector pTS543 to yield strains AL027, AL028 and AL029. Strain TS747 was further mutated to inactivate RadA splicing ($RadA^i$) (AM030), followed by reintroduction of a PolB variant via ectopic expression to yield strain AM033.

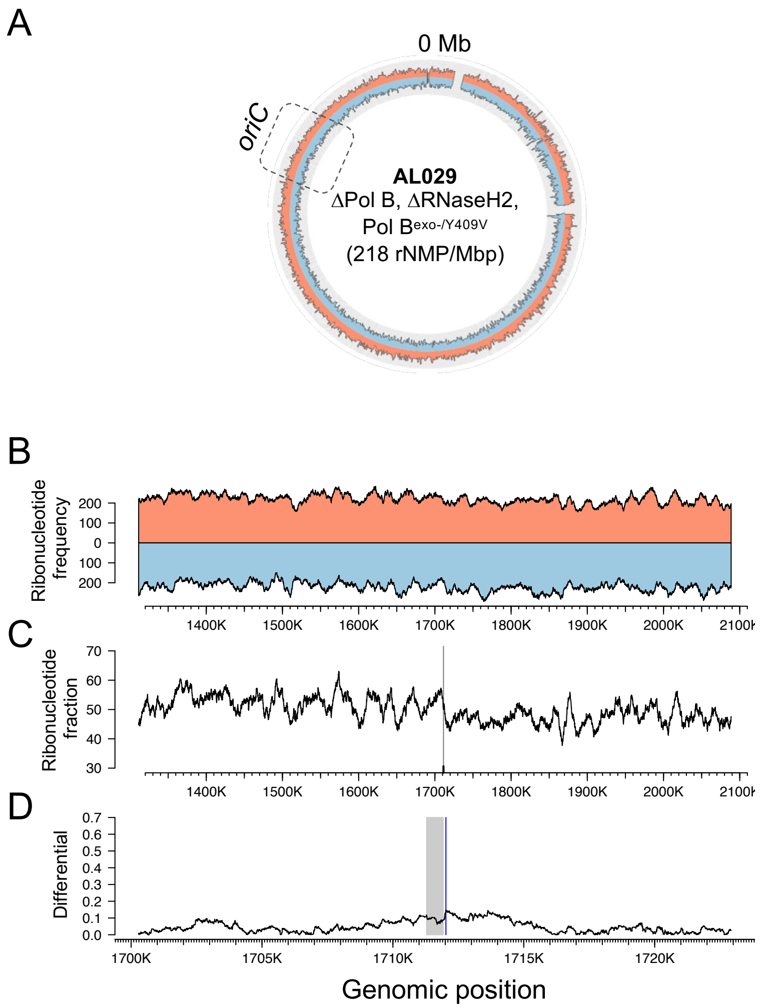


Supplemental Figure 5.RADAR-seq plot for (A) AL029 (ΔRNaseHII/ΔPolB /pTS543 PolB$^{exo-/Y409V}$). Grey box outlines the predicted origin of replication (*oriC*). (B) Ribonucleotide frequency, obtained from RADAR-seq analysis of AL029, near the putative origin of replication (C) The fraction of ribonucleotides found on the top strand is displayed in the genomic region surrounding the putative origin of replication (vertical line). (D) Precise genomic location in which ribonucleotides accumulate at the *oriC* determined by locating the position with maximal value of differential (M&M). Grey area corresponds to the predicted origin of replication and blue vertical bar is the highest differential.

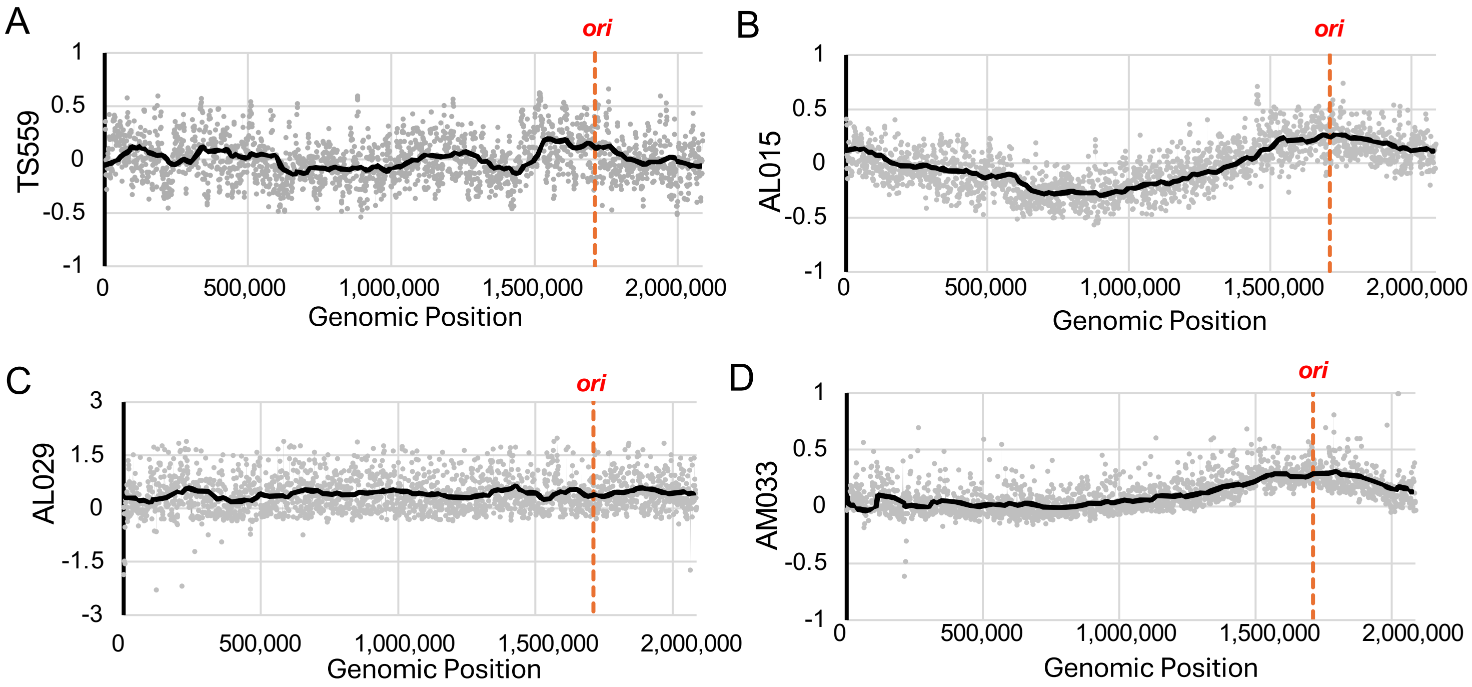


Supplemental Figure 6. MFA plots for (A) WT (TS559), (B) AL029, (C) AL015, and (D) AM033. MFA data for TS559 and AL015 were previously generated (31).

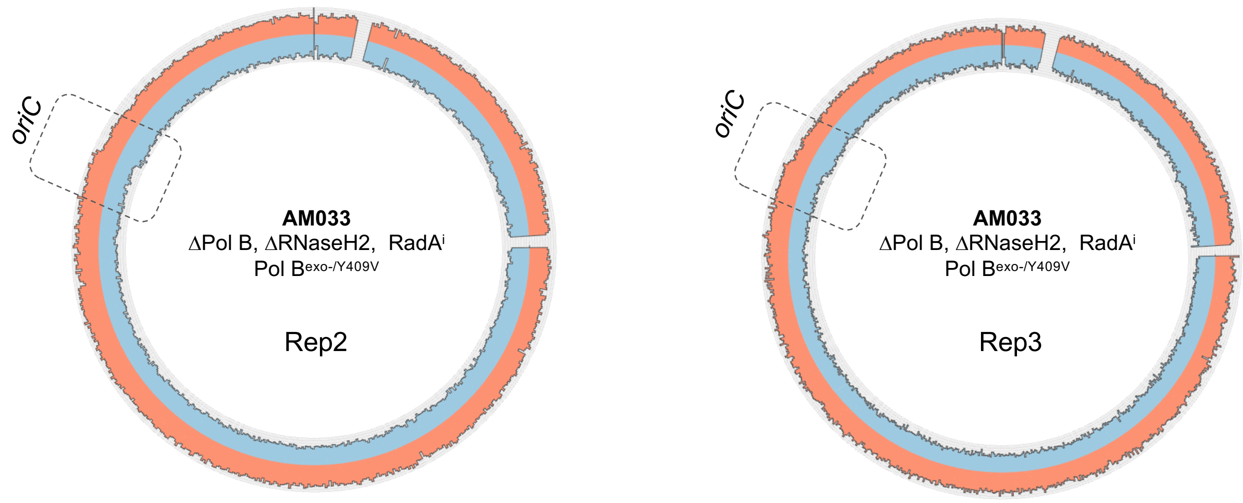


Supplemental Figure 7. RADAR-seq plots for AM033 (ΔRNaseHII/ΔPolB/RadA$^{i}$ + pTS543 PolB$^{exo-/Y409V}$) replicates. Grey box outlines the predicted origin of replication (*oriC*).

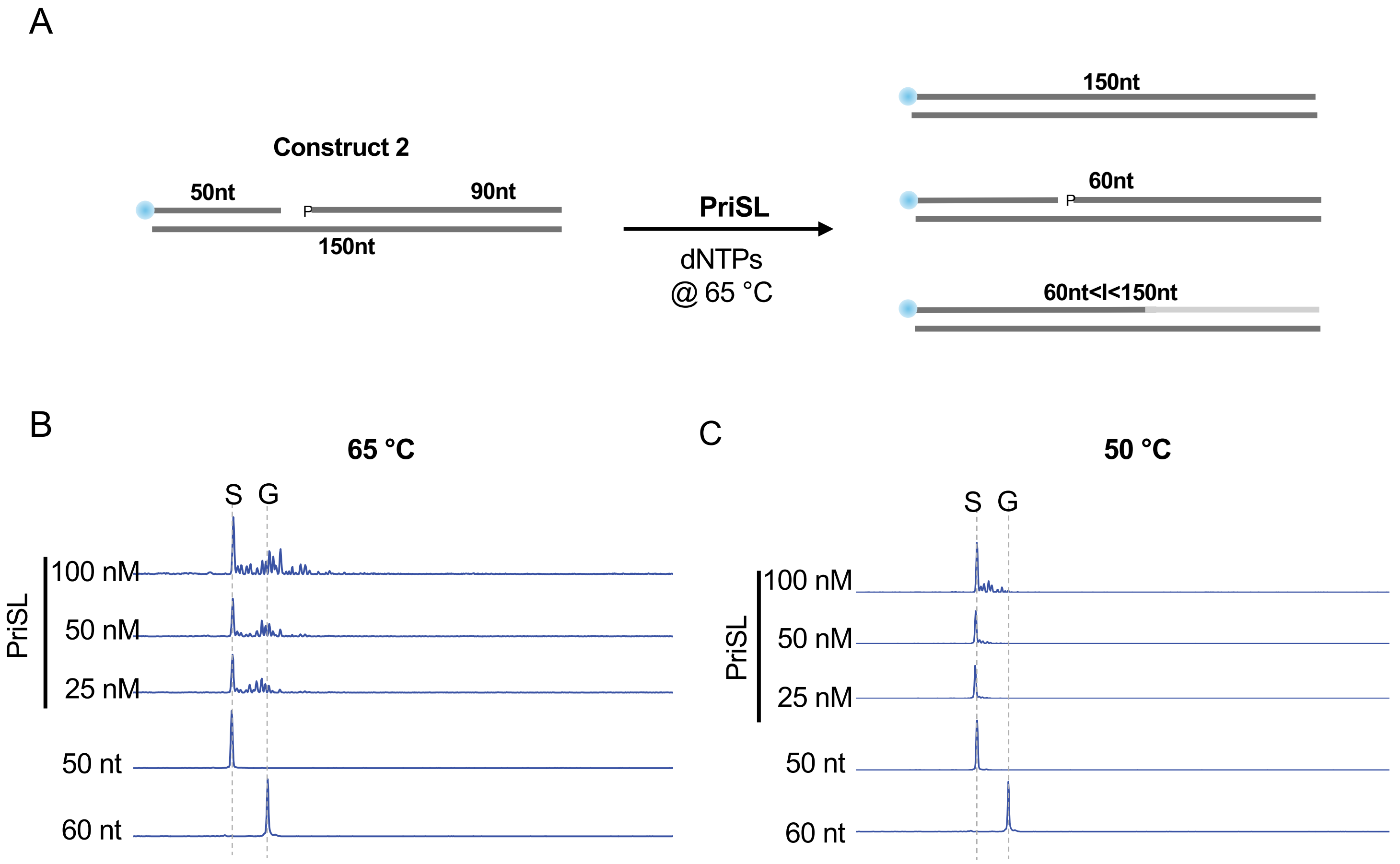


Supplemental Figure 8. Strand displacement activity of *T. kodakarensis* PriSL (A) Schematic of CE-based strand displacement assay where a 50 nt 5'-FAM labeled primer and 90 nt 5'-phosphate containing oligo are annealed to a 150 nt template and incubated with varying concentrations of PriSL at 65 °C and 50 °C for 3 mins. CE traces of PriSL strand displacement products for (B) 65 °C and (C) 50 °C incubation. (S) corresponds to 50 nt substrate, (G) corresponds to 60 nt gap product.

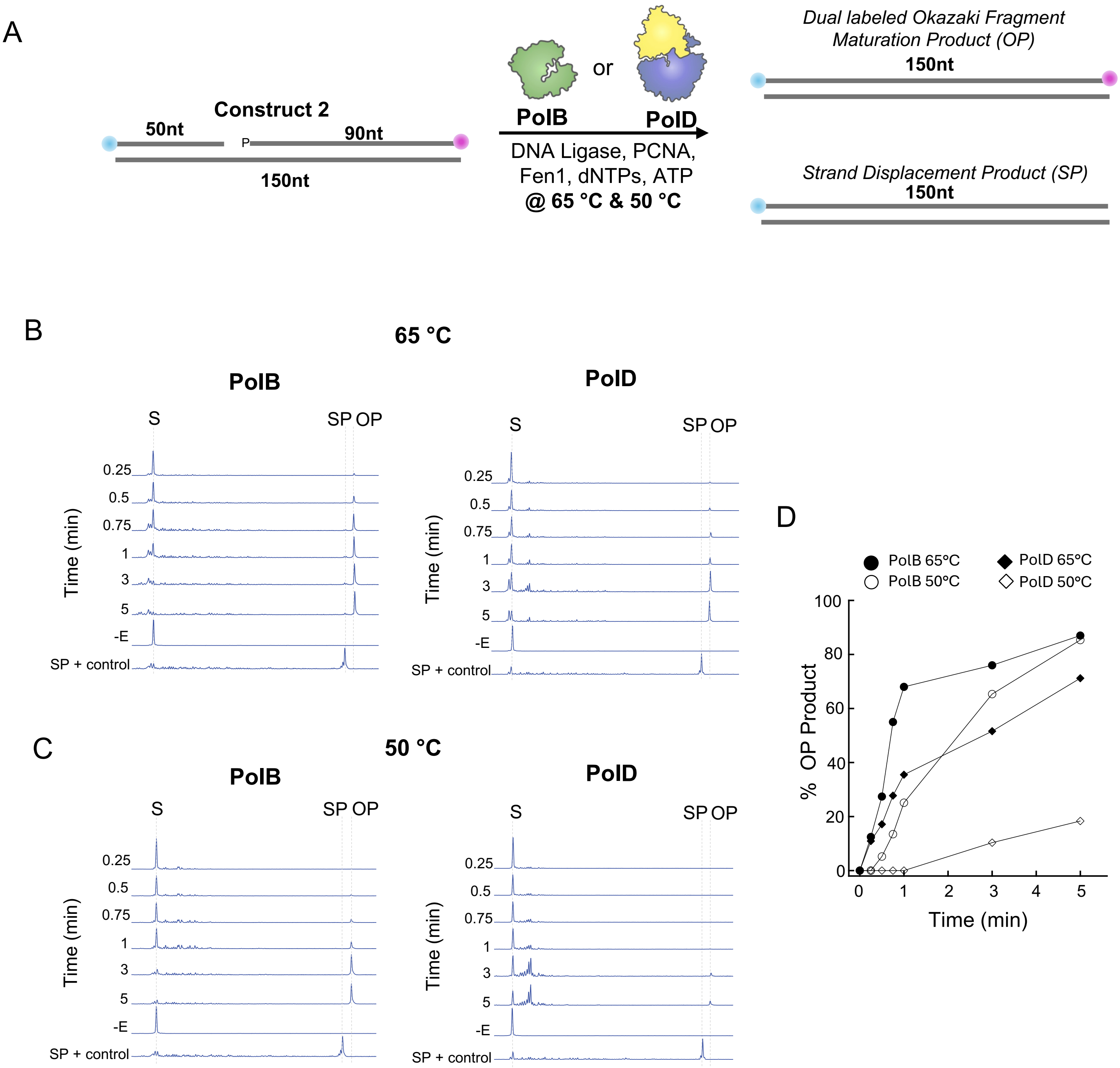


Supplemental Figure 9. *In vitro* capillary electrophoresis Okazaki fragment maturation assay. (A) Schematic of the CE-based Okazaki fragment maturation assay where a 50nt 5'-FAM primer and a 90nt 5' monophosphate 3'-Cy3 downstream all DNA oligo are annealed to a 150 nt template and incubated with Fen1, PCNA, DNA Ligase, dNTPs, ATP and either PolB or PolD at 65 or 50 °C for various time points and quenched with EDTA followed by CE analysis. CE traces from Okazaki fragment maturation time courses for PolB and PolD at (B) 65 or (C) 50 °C, where OP = Okazaki fragment maturation product, SP = strand displacement product, and SP+control is a CE trace for polymerase strand displacement reaction. (D) % Product of OP formed during Okazaki fragment maturation time courses.

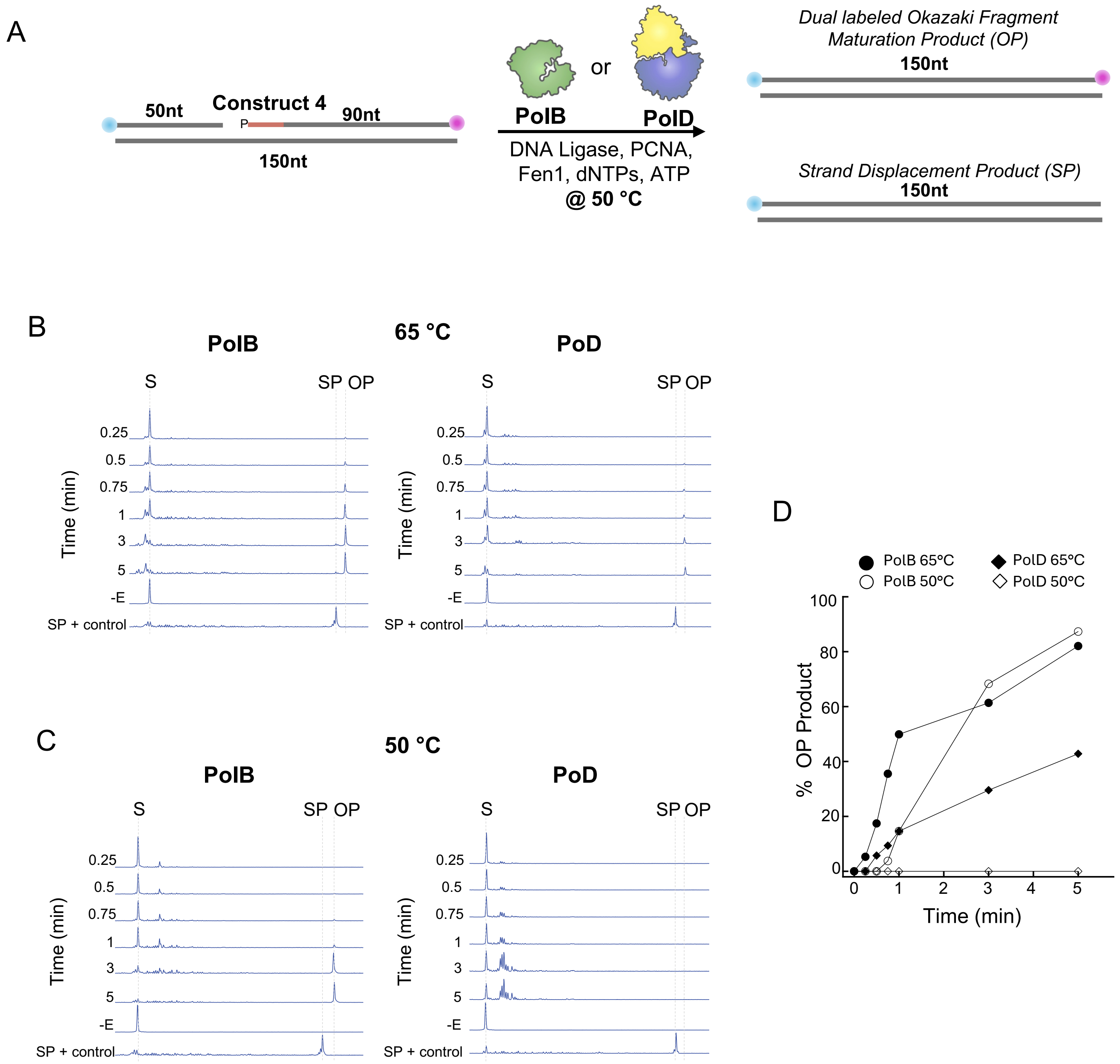


Supplemental Figure 10. *In vitro* capillary electrophoresis Okazaki fragment maturation assay. (A) Schematic of the CE-based Okazaki fragment maturation assay where a 50nt 5'-FAM primer and a 90nt 5' monophosphate RNA-DNA chimera 3'-Cy3 downstream oligo are annealed to a 150 nt template and incubated with Fen1, PCNA, DNA Ligase, dNTPs, ATP and either PolB or PolD at 65 or 50 °C for various time points and quenched with EDTA followed by CE analysis. CE traces from Okazaki fragment maturation time courses for PolB and PolD at (B) 65 or (C) 50 °C, where OP = Okazaki fragment maturation product, SP = strand displacement product, and SP+control is a CE trace for polymerase strand displacement reaction(D) % Product of OP formed during Okazaki fragment maturation time courses.

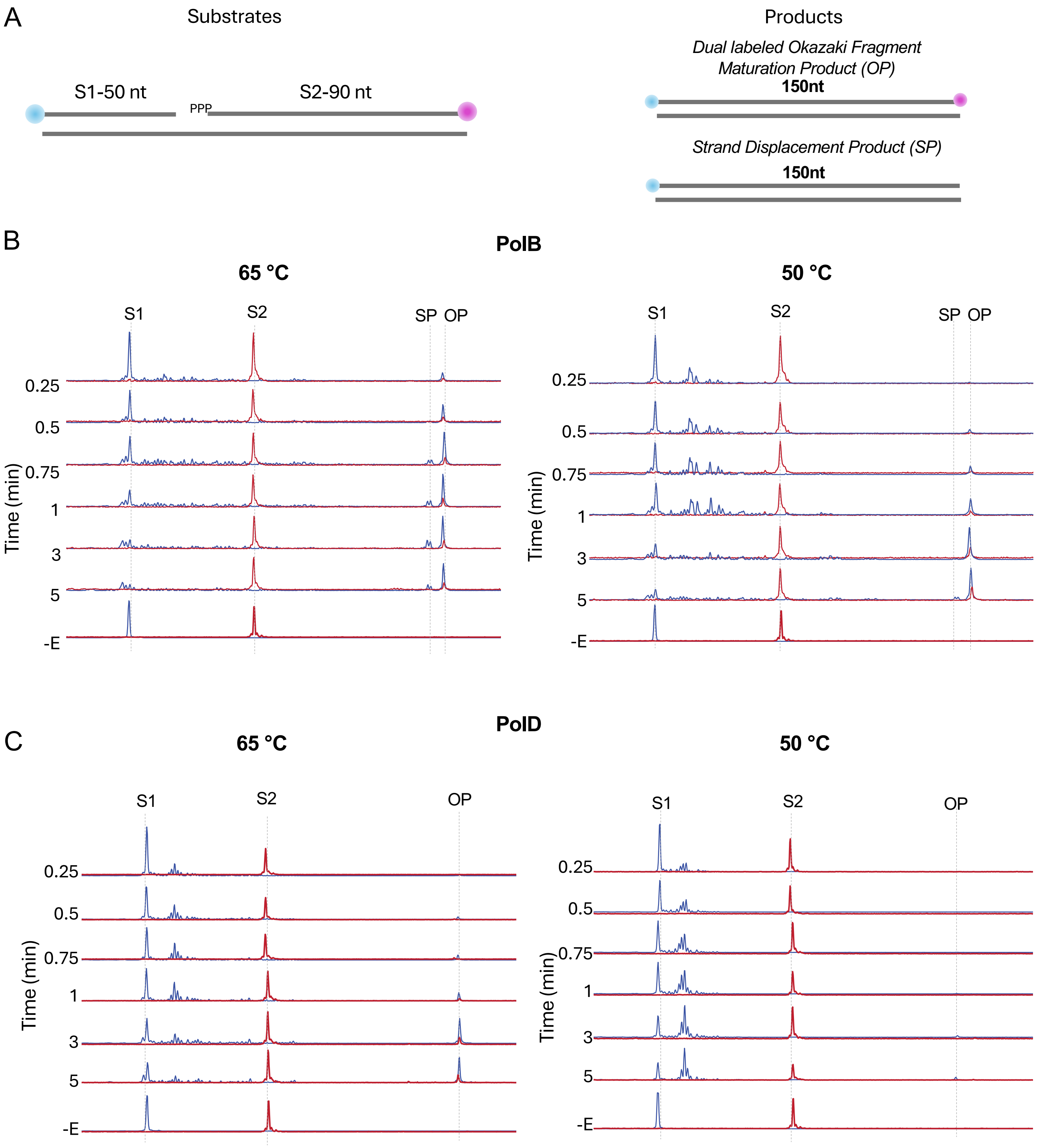


Supplemental Figure 11. Dual-labeled Okazaki fragment maturation CE-based assay. (A) Schematic of the starting substrate and products formed during the CE-based Okazaki fragment maturation assay. CE traces displaying 5'FAM labeled primer (blue) and 3'Cy3 labeled downstream strand (red) during (B) PolB and (C) PolD Okazaki fragment maturation at 65 °C and 50 °C, where a dual labeled FAM/Cy3 peak appears for the Okazaki Maturation product (OP) and not the strand displacement product (SP).

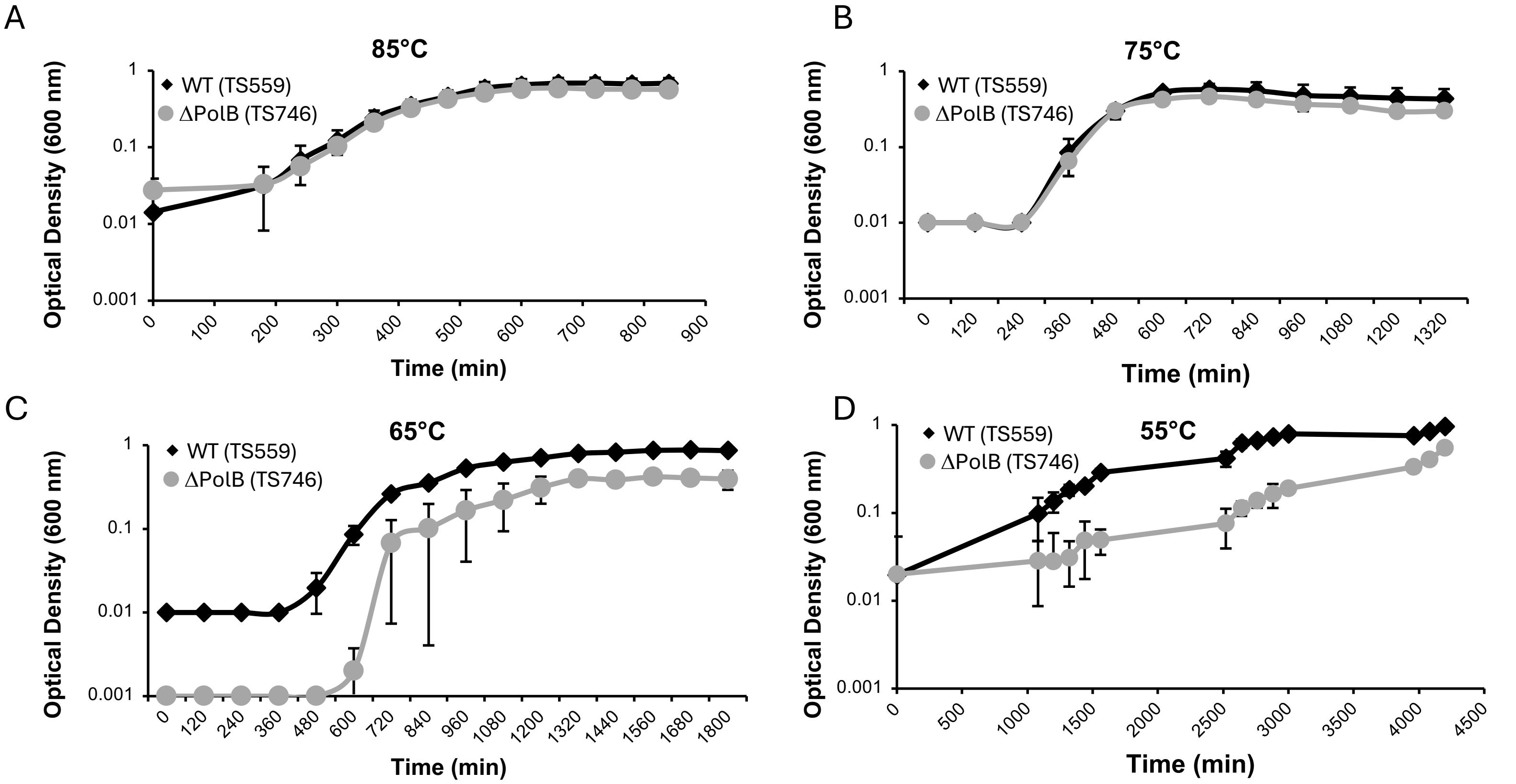


Supplemental Figure 12. Growth curves of WT (TS559) (◆) and ΔPolB (TS746) (●) *T. kodakarensis* strains at (A) 85 °C and (B) 75 °C, (C) 65 °C, and (D) 55 °C.
